# Interfacing CuO, CuBi_2_O_4_, and protective metal oxide layers to boost solar-driven photoelectrochemical hydrogen evolution†

**DOI:** 10.1039/d4dt02738h

**Published:** 2024-12-04

**Authors:** Cathal Burns, Owen Woodford, Susanna L. Stephens, Muhammed Rishan, Linsey Fuller, Shafeer Kalathil, Elizabeth A. Gibson

**Affiliations:** a Faculty of Health and Life Sciences, Department of Applied Sciences, Northumbria University Newcastle NE1 8ST UK shafeer.kalathil@northumbria.ac.uk; b Energy Materials Laboratory, Chemistry, School of Natural and Environmental Sciences, Newcastle University Newcastle upon Tyne NE1 7RU UK elizabeth.gibson@newcastle.ac.uk; c Procter & Gamble Innovation Centre Whitley Road Newcastle upon Tyne NE12 9BZ UK

## Abstract

This article reports the development of CuO|CuBi_2_O_4_ photocathodes stabilized by protective layers of TiO_2_, MgO, or NiO, with Pt or MoS_2_ nanoparticles serving as co-catalysts to facilitate H_2_ evolution. Most notably, this work demonstrates the first application of MgO as a protection/passivation layer for photocathodes in a water-splitting cell. All configurations of photocathodes were studied structurally, morphologically, and photoelectrochemically revealing that CuO|CuBi_2_O_4_|MgO|Pt photocathodes achieve the highest stable photocurrent densities of −200 μA cm^−2^ for over 3 hours with a Faradaic efficiency of ∼90%. Bias-free tandem water splitting was then performed by pairing this photocathode with a dye-sensitized TiO_2_ photoanode, producing H_2_ from neutral water without an external bias. This paper demonstrates key stability findings and proposes the use of spin-coated MgO, TiO_2_, and NiO as feasible earth-abundant protective materials to aid in the formation of a cheap and scalable tandem water splitting system. Charge transfer dynamics have also been probed by combining spectroelectrochemistry and *in situ* transient absorption spectroscopy.

## Abstract 2

This article reports the development of CuO|CuBi_2_O_4_ photocathodes stabilized by protective layers of TiO_2_, MgO, or NiO, with Pt or MoS_2_ nanoparticles serving as co-catalysts to facilitate H_2_ evolution.

## Graphical abstract

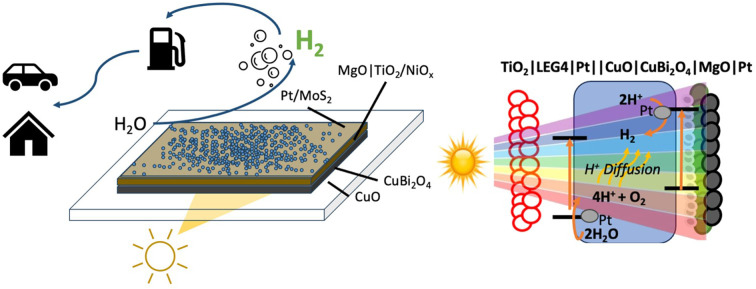

## Introduction

To enable the transition to a circular carbon economy and to meet global climate goals, clean and sustainable energy sources need to be adopted urgently. Of these potential sources, green H_2_ has emerged as an important fuel due to the high energy within the H–H bond. A promising approach for sustainable H_2_ production is photoelectrochemical (PEC) water splitting.^2,3^ PEC water splitting utilises solar energy to drive O_2_ and H_2_ evolution reactions (OER/HER) from water using photoelectrodes, assembled from photoactive materials coupled to co-catalysts, in a wired system. When photons of sufficient energy are absorbed, electrons within the semiconductors acquire enough energy to cross the band-gap, generating excited electron–hole pairs. Different configurations of PEC systems are possible, including single photoelectrode and tandem devices. PEC efficiency is affected by numerous factors such as light absorption efficiency, charge mobility in the semiconductors, charge transfer between semiconductors, co-catalysts and electrolytes, as well as the reaction kinetics.^4^

**Fig. 1:**
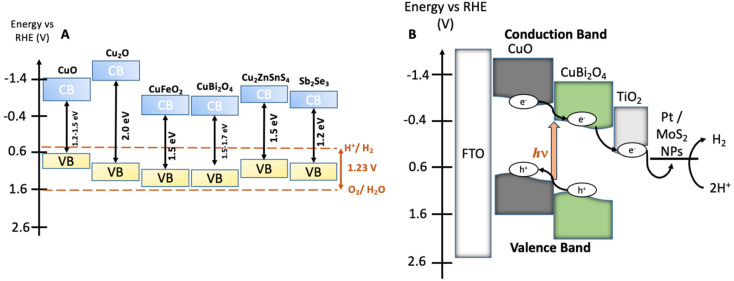
(A) Energy diagram showing the valence bands (VB), the conduction bands (CB), and the bandgaps of various earth abundant metal oxide materials which have been commonly employed as photocathodes or photoanodes in PEC water splitting cells.^1^ The water oxidation and proton reduction potentials are also shown as dashed lines. (B) Estimated band structure of a CuO|CuBi_2_O_4_|TiO_2_|Pt/MoS_2_ photocathode. All energies are given *vs*. RHE reference electrode at pH 7.2.

An extensive number of photoactive materials have been reported for efficient and stable H_2_ evolution. Electrons liberated by photocathodes are used to reduce aqueous protons forming H_2_. In p-type semiconductors, excitation with light leads to the movement of the majority carriers (holes) through the material, while the minority carriers (electrons) travel to the surface where the HER occurs. A fairly narrow band gap (E_g_) is necessary to absorb visible sunlight. The most efficient semiconductors used in photocathodes for HER include p-type Si, GaP, InP, CdTe, CuIn_*x*_Ga_1*x*_Se_2_, Cu_2_ZnSnS_4_, CuGa_3_Se_5_ and CuGaSe_2_.^5^ The photovoltages of these materials remain low (<0.75 V). The minimum potential required to split water molecules under neutral pH conditions is 1.23 V. Photocathodes which display high photovoltages will allow for efficient tandem devices for bias-free solar water splitting.^6^ Additional challenges with many good photovoltaic semiconductors include the scarcity of In and Ga, heavy elements such as Cd are toxic, synthesis of photovoltaic-quality Si (high crystallinity and purity) is energy intensive, and Si and many metal sulfides are unstable under photocatalytic water splitting conditions.^7^ Therefore, there is a requirement to study transition metal oxide materials composed of earth abundant and non-toxic elements that can be used as photoelectrodes for water splitting (Fig. 1A).^8,9^ Most Cu-based transition metal oxides have a conduction band edge that is more negative than the HER potential, which ensures light-driven proton reduction is thermodynamically favourable.^1^ The valence band (VB) of Cu-oxides is higher than most other transition metal oxides, leading to narrower band gaps and more effective solar light absorption. In CuO, for example, the valence band is composed of Cu 3d orbitals coupling to O 2p states.^10^ CuO possesses a desirable bandgap (∼1.2–1.5 eV) for PEC HER with valence and conduction band edges straddling the proton reduction potential. However, CuO is limited in terms of practicality due to poor stability in aqueous and acidic media as the reduction potential of Cu^II^ lies within the band gap of the semiconductor and charge mobility is poor.^11^

To overcome this and improve performance, hybrid structures and heterojunctions have been employed.^12,13^ Ternary Cu-based oxides such as CuBi_2_O_4_ provide alternative opportunities. CuBi_2_O_4_ has a bandgap of 1.5–1.7 eV with an onset potential of *ca.* 1.55 V *vs*. RHE which is an extremely promising quality for applications as a photocathode in a tandem device.^14–16^ Despite these promising qualities, photo corrosion is still a major issue. Within CuBi_2_O_4_, photo corrosion occurs because of trapped photoelectrons within the conduction band (CB), which is largely Cu 3d in character.^17^ As is the case for the binary Cu oxides, protective methods are necessary to utilise these materials for long term applications. For example, Grätzel *et al.* have conducted extensive work on TiO_2_ protected Cu_2_O photocathodes. These have been applied in PEC H_2_ evolution systems with reported photocurrent densities of 10 mA cm^−2^ for up to 50 hours.^18^

A method of protecting the photocathodes from water is needed to prevent this degradation.^19^ TiO_2_ has frequently been employed as a protective material. Atomic layer deposition (ALD) is often recommended to enable a high-level of control to build a thin and homogeneous metal oxide coating.^18,20^ However, the precursors of such materials for ALD are expensive, often toxic, and the procedure requires specialist equipment. Previous reports of CuBi_2_O_4_ in the literature show that inkjet printing of can be used to fabricate photocathodes which produce ∼0.12 mA cm^−2^ with a photocurrent onset potential of up to 1 V *vs*. RHE.^21^ In 2022, an alternative approach was trialled using inverse opal CuBi_2_O_4_.^22^ This high surface area electrode produced 2.95 mA cm^−2^ with H_2_O_2_ as an electron scavenger and a stability of 2 hours. The high surface area inverse opal morphology to increase photocurrent density would be complimentary with our liquid-phase synthesis. We apply our photocathodes to carry out overall water splitting without the H_2_O_2_ scavenger, forming a cleaner system. Intensity-modulated photocurrent spectroscopy (IMPS) studied charge transfer dynamics within CuBi_2_O_4_. CuBi_2_O_4_ has previously been hybridized with CuO to produce photocurrents of 1.23 mA cm^−2^ at 0 V *vs*. RHE and a reported 2 hours stability.^23^ In this example, the fabrication methods used involve a hydrothermal synthesis of CuO onto FTO in an autoclave, followed by up to 15 layers of spin-coating from a precursor solution and annealing to form CuBi_2_O_4_. This method produced compact layers of each material. The advantages of our approach are importantly the scaleability and adaptability of spray pyrolysis and co-precipitation synthesis. Co-precipitation can be used to synthesise alternative metal oxides which leads to future avenues of exploration. We also delve deeper into protective strategies and fundamental spectroscopic and electrochemical analysis.

In our study, we introduce a scalable liquid-phase preparation of CuO|CuBi_2_O_4_ heterojunction photocathodes and investigated three potential materials for protecting the electrodes (TiO_2_, MgO, and NiO). This work represents the first reported application of MgO as a passivating layer for a photocathode PEC water-splitting system. This discovery can allow researchers to expand upon the current state-of-the-art viable materials that allow long term PEC stability. CuBi_2_O_4_ has was chosen to promote charge separation at the interface with CuO.^6,24,25^ The decreased rate of recombination facilitated more efficient HER at the electrode–electrolyte interface. In a typical metal–oxide based PEC system, the HER kinetics are relatively slow in comparison to the rate of recombination. Therefore, an electron transfer cascade encourages electrons to go to the catalyst surface rather than recombining with holes in the semiconductors. The fabrication techniques such as automated co-precipitation of CuBi_2_O_4_ powder, blade-coating of CuBi_2_O_4_ paste, spray pyrolysis of CuO films, spin-coating the passivating materials, and drop-casting the co-catalysts, are all suitable for large-scale production.

To carry out efficient HER at the photocathode–electrolyte interface, colloidal platinum (Pt) particles were deposited to act as a benchmark HER co-catalyst.^26^ The role of the co-catalyst is to assist in the reduction of aqueous protons when added to the surface of the CuO|CuBi_2_O_4_ photocathodes, improving the stability and efficiency. The scarcity and cost of Pt makes it preferable to find earth-abundant alternatives. MoS_2_ has emerged as one of the most promising candidates for efficient HER at a fraction of the cost of Pt.^27^ In this paper, we explore MoS_2_ as a feasible alternative co-catalyst. The stabilised photocathodes were paired with dye-sensitized TiO_2_ photoanodes^28^ to demonstrate tandem bias-free PEC water splitting and H_2_ evolution was quantified using gas chromatography (GC).

The photocathode architectures were studied using a combination of electrochemistry and optical spectroscopy to understand the effects of these coatings on the CuO|CuBi_2_O_4_ photocathodes, specifically we employed spectroelectrochemistry and transient absorption spectroscopy (TAS). These techniques allowed the charge dynamics and redox processes within the material to be studied on ultrafast timescales (fs–μs), providing valuable insight into how the addition of each layer impacted electron transfer/charge separation mechanisms and electrode stability.

## Results and discussion

### Characterization of CuO|CuBi_2_O_4_ photocathodes

**Fig. 2:**
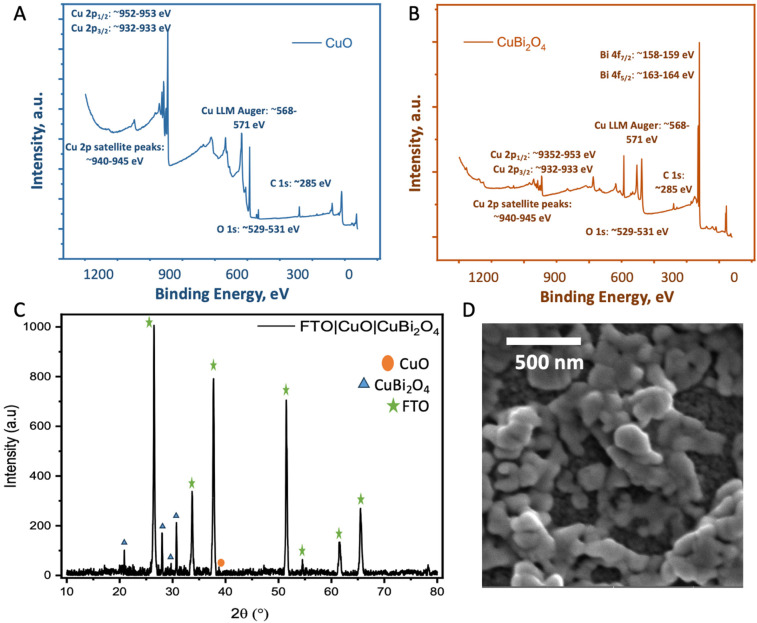
Physiochemical characterization. (A and B) XPS survey scans of CuO showing Cu 2p, O 1s and CuBi_2_O_4_ (top) showing the Cu 2p, O 1s and Bi 4f peaks. (C) XRD of FTO|CuO|CuBi_2_O_4_. (D) SEM image of the CuO|CuBi_2_O_4_ electrodes. The parameters of the SEM images are given in Fig. S5.†

The synthesis of CuBi_2_O_4_ was carried out in an automated co-precipitation reactor (Experimental section). X-ray diffraction (XRD) was carried out to characterize films of CuBi_2_O_4_ on FTO substrates and CuBi_2_O_4_ powder (Fig. S1†) and the diffraction patterns were compared with previously reported data in the literature. The match with the reference diffraction pattern (kusachiite, PDF no. 00-042-0334) confirmed that the synthesis method was successful. To enhance the photocurrent from CuBi_2_O_4_ through more efficient light absorption and charge separation, it can be coupled with CuO to form a heterojunction.^24^ This is due to the band alignment portrayed in Fig. 1B which promotes electron transfer towards the HER catalyst at the interface, reducing recombination. CuO deposition was achieved by spray pyrolysis^29^ from a Cu(NO_3_)_2_ solution onto FTO substrates at 450 °C. A layer of CuBi_2_O_4_ was then blade coated and annealed onto the CuO surface to form the heterojunction. X-ray photoelectron spectroscopy (XPS) survey scans were carried out on CuO and CuBi_2_O_4_ (Fig. 2A, B and S2, 3†). XPS also confirmed that the VB for CuO was 4.88 eV *vs*. vacuum (Fig. S4, S5 and Table S1†), with deeper electronic states of Cu 2p_3/2_ Cu 2p_3/2_ at 952–953 eV (along with satellite peaks at 940–945 eV) and O 1s orbitals present at 529–531 eV in the survey scan. Additional peaks at ∼570 eV were also present. These are consistent with Cu (LLM) Auger peaks, indicating the presence of Cu^2+^. These peaks were also found in CuBi_2_O_4_ (Fig. S2†). The VB of CuBi_2_O_4_ was found to be ∼5.9 eV *vs*. vacuum (Fig. S4†), with deeper electronic states of Cu 2p_3/2_, 2p_1/2_, O 1s, Bi 4f_7/2_, Bi 4f_5/2_ (at 158–159 and 163–164 eV, respectively) orbitals all present in the survey scan (Fig. S3†). These results are consistent with reported XPS values in the literature for CuO^30^ and CuBi_2_O_4_.^17^ The XRD pattern for the thin films confirmed the successful formation of the CuO|CuBi_2_O_4_ electrode, with peaks present from CuBi_2_O_4_, CuO, and FTO (Fig. 2C). UV-visible spectroscopy was used to determine the band-gap of each material (Fig. S6†). The Tauc plots show that CuBi_2_O_4_ and CuO have very similar band-gaps (both ∼1.5 eV). The VB positions of both materials, together with the measured band-gap indicate a favourable band alignment for photoinduced charge separation (Fig. 1B).^17^ The absorbance spectrum showed that films of CuBi_2_O_4_ absorb up to 600 nm, consistent with a bandgap of 1.5 eV. Fig. S6A† shows the reflectance spectra of CuO and CuO|CuBi_2_O_4_ films. The addition of CuBi_2_O_4_ increased the percentage of visible light absorbed by the electrode. The morphology of CuO|CuBi_2_O_4_ electrodes was also characterized using scanning electron microscopy (SEM) (Fig. 2D and S7†). SEM showed the presence of nanoparticles of various sizes with an average diameter of around 100 nm. SEM-EDS (Fig. S8†) was also carried out confirming the presence of C, O, Bi, Cu, Sn, and F atoms, which is consistent with CuBi_2_O_4_ on the FTO substrate.

**Fig. 3:**
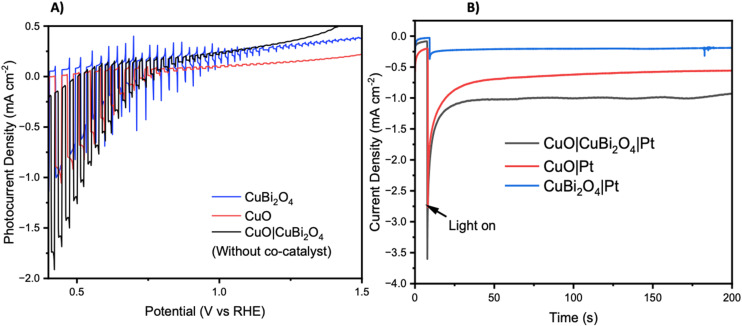
Photoelectrochemistry of unprotected photocathodes. (A) Chopped light linear sweep voltammetry of CuO, CuBi_2_O_4_, CuO|CuBi_2_O_4_ at 10 mV s^−1^ scanning from positive to negative. (B) Chronoamperometry under constant illumination at 0.4 V *vs*. RHE. Photo experiments were carried out using a Xe arc lamp calibrated to 1 sun (AM1.5G, 100 mW cm^−2^).

Chopped-light linear sweep voltammetry and chronoamperometry experiments were tested on each material. Chopped-light linear sweep voltammetry of CuO, CuBi_2_O_4_, and CuO|CuBi_2_O_4_ (Fig. 3A) revealed that CuBi_2_O_4_ produced the smallest photocurrents but showed the highest stability, as expected. When chopping the light on and off, spikes are observed in the CuBi_2_O_4_ voltammogram, particularly present at 0.7 V *vs*. RHE. These indicate charging and discharging at the surface, along with trap states existing at the electrode–electrolyte interface. CuO films alone displayed high instability under the experimental conditions with a large dark current occurring below 0.6 V *vs*. RHE, a clear sign of degradation. This degradation also clearly impacts the photocurrent observed from the CuO across the scan. Chopped light chronoamperometry was also carried out at a fixed potential of 0.4 V *vs*. RHE to compare the photocurrent densities of CuO and CuO|CuBi_2_O_4_. CuO displays a large dark current of −0.5 mA cm^−2^ with an initial photocurrent of *ca.* −1 mA cm^−2^ which diminishes to −0.25 mA cm^−2^ after 2 minutes. The dark current for the CuO|CuBi_2_O_4_ electrode was relatively smaller, which was accompanied by an initial photocurrent density of −1.5 mA cm^−2^. Stability over a 2-minute period was shown to increase with *ca.* −1.2 mA cm^−2^ remaining after 2 minutes. These results demonstrate the effectiveness of pairing CuO with CuBi_2_O_4_. Varying the thickness of the CuBi_2_O_4_ was not found to increase the observed photocurrent. The best performance was achieved using a single layer (*ca.* 500 nm) of CuBi_2_O_4_ in both instances (Fig. S9†). This is likely due to enhanced recombination due to the increasing diffusion length occurring with thicker CuBi_2_O_4_ layers. Chronoamperometry was also performed on CuO, CuBi_2_O_4_, and CuO|CuBi_2_O_4_ with Pt co-catalysts added to the surface. Again, the results (Fig. 3B) show the enhancement of photocurrent generation when CuO and CuBi_2_O_4_ are interfaced.

### Protecting the photocathodes

**Fig. 4:**
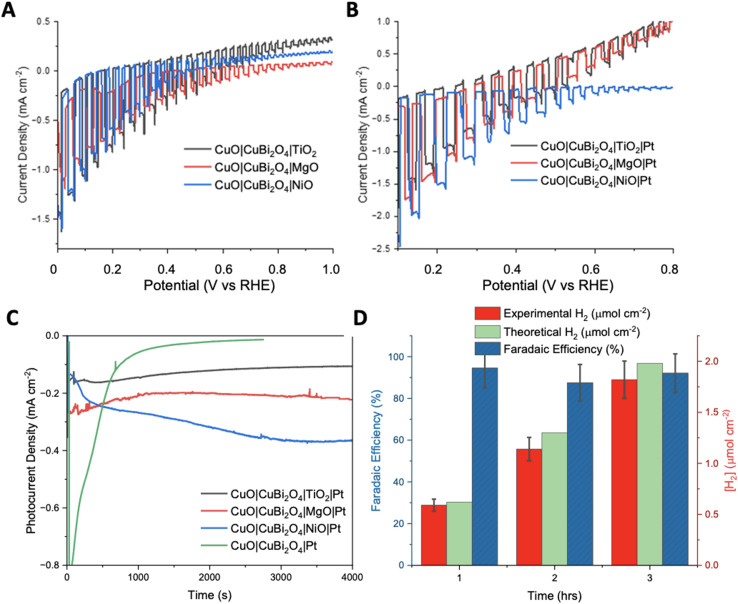
Photoelectrochemistry of protected photocathodes. (A) Chopped light linear sweep voltammetry comparing the effect of each protecting layer on photocurrent production. (B) Chopped light linear sweep voltammetry showing the addition of Pt. Illumination was achieved using a Xe arc lamp calibrated to 1 sun (AM1.5G, 100 mW cm_−2_). (C) Chronoamperometry of each protected photocathode and the unprotected photocathode with Pt co-catalyst. The bare electrode, TiO_2_ protected and MgO protected were carried out at 0.4 V and the NiO protected photocathode was carried out at 0.3 V *vs*. RHE. All experiments were under constant illumination. (D) Experimental and theoretical H_2_ accumulation after each hour of chronoamperometry (1–3 hours) at 0.4 V *vs*. RHE under constant illumination with a Xe arc lamp calibrated to 1 sun (AM1.5G, 100 mW cm^−2^) with CuO|CuBi_2_O_4_|MgO|Pt as the working electrode, 3.5 M KCl Ag/AgCl as the reference and Pt wire as the counter electrode. Faradaic efficiencies for each hour are also displayed in blue. Error bars were calculated from the standard deviation (*n* = 3).

To inhibit photo corrosion, three different transparent metal oxides that are stable under illumination in aqueous conditions were used to pacify the surface of CuO|CuBi_2_O_4_. A thin layer of TiO_2_, MgO, and NiO were trialled separately. Each material was deposited using a simple spin-coating method followed by annealing at 450 °C. TiO_2_ and NiO offer semi-conductive (n-type and p-type, respectively), semi-transparent transparent, and stable surface protection from the aqueous protons. MgO also offers transparency and stability but is an insulator.^31^ Chopped-light linear sweep voltammetry using the CuO|CuBi_2_O_4_|TiO_2_/NiO/MgO electrodes suggested that that all three inhibited the photo corrosion, with a large decrease in dark current observed in each case (Fig. 4A). However, much lower photocurrent density was also observed compared to CuO|CuBi_2_O_4_, which can be attributed to a combination of reduced corrosion, the TiO_2_/NiO/MgO coating blocking some of the pores of the CuBi_2_O_4_, and, if the layer becomes too thick, inhibited charge flow to the catalytic sites. Chronoamperometry under constant illumination at 0.4 V *vs*. RHE showed a stable current density of −0.1 mA cm^−2^ for over 3 hours for the TiO_2_ coated electrode. For MgO, photocurrent density was surprisingly enhanced in comparison to TiO_2_, with a steady current density of −0.2 mA cm^−2^ at 0.4 V *vs*. RHE. These results suggest that MgO pacifies the surface more effectively than TiO_2_, while permitting charge transfer. For NiO samples, an optimum photocurrent density as large as −0.3 mA cm^−2^ at 0.3 V *vs*. RHE was attained.

The SEM images (Fig. S10 and S11†) of the CuO|CuBi_2_O_4_|NiO sample showed a uniform coverage of the CuO|CuBi_2_O_4_ nanoparticles. The surface of the electrode appears to be much thicker than TiO_2_ or MgO samples (Fig. S12–S15†), with cracks apparent on the surface. These cracks are consistent with NiO blocking layers deposited on a metal oxide surface (FTO), which were previously reported for applications in p-type dye-sensitized solar cells.^32^ SEM-EDS confirmed that the NiO layer was much thicker than MgO or TiO_2_ with a 17.53% weight for Ni (Fig. S10†). Despite the increased atomic mass of Ni in comparison to Mg or Ti, this difference in abundance is consistent with a thicker layer. SEM images of CuO|CuBi_2_O_4_|TiO_2_ photocathodes, shown in Fig. S12,† show a uniform coating of nanoparticles. The nanoparticle size ranged between hundreds of nm down to <100 nm. SEM-EDS confirmed the presence of Ti atoms on the surface. The morphology and particle sizes for the CuO|CuBi_2_O_4_|MgO sample were similar to the TiO_2_ coated sample (Fig. S15†), which is consistent with a thin coating of the respective transparent metal oxide on surface of the larger CuBi_2_O_4_ particles on the electrolyte-facing surface of the electrode. SEM-EDS confirmed the presence of Mg on the surface of the electrode, with a calculated weight percentage of Mg = 0.11% (Fig. S14b†). Cross sectional SEM was performed on each configuration (Fig. S16†). The images show an average thickness of CuO to be *ca.* 1.5 μm and CuBi_2_O_4_ to have an average thickness of *ca.* 500 nm. The passivating materials were unable to be directly visualized as individual layers. However, a change in thickness of the top mesoporous CuBi_2_O_4_ layer was observed for each material. For MgO, the top layer was passivated by an additional 0.5–1 μm which could be attributed to both MgO and Pt infiltrating through the porous structure. For TiO_2_, a similar effect is observed within the top layer. This time very little changes were observed. This suggests that the TiO_2_ and Pt formed a much thinner layer when infiltrated (likely a few hundred nm). Lastly, with NiO the thickness of the top layer was found to be *ca.* 1.2 μm meaning that as expected and observed in the top-view SEM, the NiO|Pt produces the thickest passivating layer.

The photocurrent density was recorded during chopped-light linear sweep voltammetry for the photocathodes with the Pt co-catalyst added. For the CuO|CuBi_2_O_4_|TiO_2_|Pt photocathodes, the addition of a Pt HER catalyst made very little difference compared to the Pt-free equivalent (Fig. 4B). A reason might be that the electrons could not effectively travel through the TiO_2_ from the CB of CuBi_2_O_4_ to the Pt catalyst on the surface. It is possible that the TiO_2_ layer was too thick to permit electron transfer to the Pt catalyst. The chronoamperometry experiments (Fig. 4D) showed that the optimum potential for a stable photocurrent was 0.4 V *vs*. RHE for TiO_2_ samples and 0.3 V for NiO samples, with a photocurrent density of *ca.* −0.3 mA cm^−2^ for >3 hours. The photocurrent density was enhanced for the CuO|CuBi_2_O_4_|NiO|Pt photocathode compared to the Pt-free sample (Fig. 4B). This suggests that electron transport through the NiO layer was permitted. For all NiO samples, the photocurrent density increased over time. A reason may be that the surface changes and a more active Ni-based catalyst is formed.

The chopped-light linear sweep voltammetry with CuO|CuBi_2_O_4_|MgO|Pt showed a promosing enhancement of the photocurrent below 0.4 V *vs*. RHE (Fig. 4B). The results suggest that the transport of electrons to the catalyst in the presence of MgO is effective. This represents the first report of the successful use of MgO as a protection layer for Cu-based photocathodes. The results demonstrate that MgO protection layers, applied *via* spin-coating, stabilised the CuO|CuBi_2_O_4_ photoactive materials, whilst also allowing the transport of electrons through the MgO to reach the Pt catalytic sites where HER can occur. The MgO layer produced the most effective and promising results at 0.4 V *vs*. RHE. This result came as a surprise due to the insulating nature of MgO, however, we have demonstrated that MgO can act as a protective layer if an adequately thin layer is used. This result agrees with the recent report by Hu *et al.*, who proposed that MgO nanoparticles integrated within CuBi_2_O_4_ can passivate the surface-trap states which can result in enhanced performance.^33^

Cyclic voltammetry (CV) was performed (without light) to compare the electrochemical characteristics of the unprotected CuO, CuBi_2_O_4_, and CuO|CuBi_2_O_4_ photocathodes (Fig. S17†). The CV of the CuO electrode contained two reduction peaks at 0.05 and −0.05 V *vs*. RHE, which correspond to the reduction of Cu^II^ to Cu^I^ followed by Cu^0^. The anodic sweep contained two oxidation peaks at 0.5 V and 1.0 V *vs*. RHE. For the CuBi_2_O_4_ electrode, a large reduction peak at −1.35 V *vs*. RHE and a small shoulder at 0 V *vs*. RHE were present. For this electrode, only one large oxidation peak was observed at 0.7 V *vs*. RHE. For the CuO|CuBi_2_O_4_ electrode, the CV contained a combination of the peaks observed with the individual semiconductors. Interestingly, protecting the photocathodes and adding the cocatalyst (CuO|CuBi_2_O_4_|MgO|Pt) significantly lowered the magnitude of the reduction peaks observed compared to the unprotected electrodes. This supports the positive impact of passivating and protecting the electrode from the electrolyte.

Following the success with the CuO|CuBi_2_O_4_|MgO|Pt electrodes, we compared the Pt catalyst with an earth-abundant MoS_2_ colloidal HER co-catalysts. Fig. S18† shows the enhancement in photocurrent observed with both co-catalyst decorated photocathodes compared to CuO|CuBi_2_O_4_|MgO sample. The results show that both Pt and MoS_2_ can use the photogenerated electrons to drive H^+^ reduction at the electrode–electrolyte interface. The addition of MoS_2_ produces a similar photocurrent density to adding Pt in the chopped LSV, suggesting that MoS_2_ could function as a viable alternative to Pt. For long term chronoamperometric experiments Pt was used exclusively as a benchmark co-catalyst. Here, our main focus was on the application of the protective materials and their capacity to facilitate charge transport to the co-catalyst.

To quantify the effectiveness of the photocathode design, H_2_ evolution was quantified using gas chromatography (GC). The MgO passivated sample with the Pt co-catalyst was chosen as it produced the highest, stable photocurrent. Fig. 4D shows the quantity of H_2_ evolved at 0.4 V *vs*. RHE under constant illumination for a 3 hours period. After 1 hour, ∼0.59 ± 0.05 μmol cm^−2^ of H_2_ had been evolved at a Faradaic efficiency of 94.5%. The 2^nd^ hour yielded ∼1.14 ± 0.15 μmol cm^−2^ of H_2_, corresponding to an 87.5% Faradaic efficiency. The 3^rd^ hour showed that the high Faradaic efficiency was maintained at 92.1% with ∼1.81 ± 0.24 μmol cm^−2^ (Tables S2 and 3†). Typically, after the 3-hour reaction period the photocurrent decreased as photodegradation began to take effect. It was observed throughout most samples that once degradation begins the photocurrent response would dissipate quickly (<5 minutes). An explanation for this is that once the electrolyte has been able to penetrate a defect in the passivation layer, the photoreduction of the Cu^II^-based materials can occur easily. The stabilising effect of the protective materials was confirmed by performing XRD pre- and post-PEC. In each case the diffractograms show no significant changes (Fig. S19–21†). This demonstrates that the crystal structure of CuO and CuBi_2_O_4_ remain stable under the conditions of the PEC reaction. This stability suggests an added resilience in the presence of the protective coatings.

To avoid the need to apply an external voltage, a tandem bias-free water-splitting system composed of a photoanode (TiO_2_|LEG4|Pt) and a photocathode (CuO|CuBi_2_O_4_|MgO|Pt) was employed to achieve efficient solar-to-H_2_ conversion. The tandem cell was configured to illuminate through the photoanode first, before reaching the photocathode. The photoanode was composed of dye-sensitized TiO_2_ (with a commercial dye, LEG4, as the photosensitizer), co-functionalised with Pt for OER. Upon illumination, the photoanode carries out water oxidation, whilst the photocathode carries out H_2_ evolution. The structure of LEG4 is shown in Fig. S22A.† The absorbance spectrum of the dyed TiO_2_ film (Fig. S22B†) shows that the films absorb mostly in the blue region of the visible spectrum, which allows the longer wavelengths to pass through to the photocathode. In this way, the tandem cell enables unassisted water splitting without additional applied bias.

**Fig. 5:**
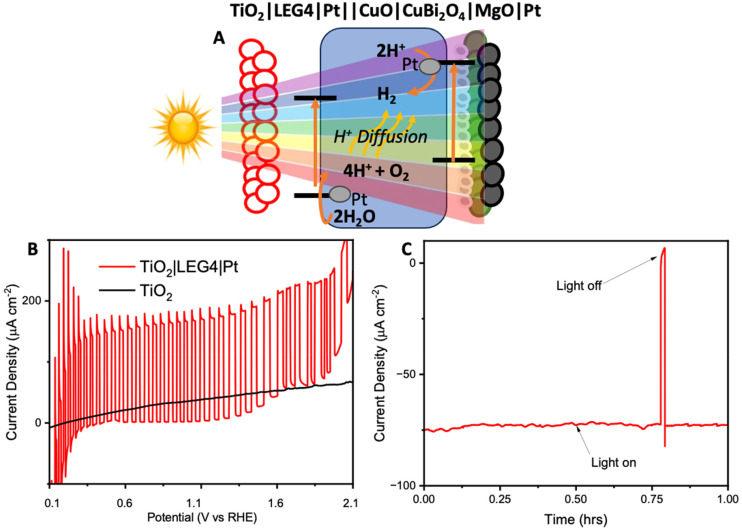
Photoelectrochemistry of the bias-free tandem. (A) An illustration of the mechanism within the tandem upon illumination. (B) Chopped light linear sweep voltammetry of TiO_2_ (black) and TiO_2_ sensitized with LEG4 and Pt (red). (C) Chronoamperometry under no bias and constant illumination with 1 sun (Xe lamp, AM1.5G, 100 mW cm^−2^) for 1 hour.

Firstly, the FTO|TiO_2_|LEG4|Pt photoanodes were tested using chopped light LSV (Fig. 5B). The results showed the photoanodes could reach photocurrent densities up to 0.15 mA cm^−2^ from 0.4 V to 1.2 V *vs*. RHE. Beyond 1.3 V *vs*. RHE, the dark current began to increase. This increase could be caused by a combination of dye oxidation and TiO_2_/Pt starting to catalyse oxygen evolution. Next, to demonstrate bias-free, pH neutral, water splitting, chronoamperometry was performed for 60 minutes using TiO_2_|LEG4||Pt||CuO|CuBi_2_O_4_|MgO|Pt, the complete tandem cell, which was illuminated through the anode. Fig. 5C shows the observed photocurrent of the tandem was 75 μA cm^−2^, with 93% of the photocurrent remaining after 60 minutes. H_2_ evolution was quantified over this period using GC. After 1 hour, 378 ± 25 nmol cm^−2^ of H_2_ had been evolved. The Faradaic efficiency of the bias-free tandem was calculated to be 58.3 ± 3.7% for 1 hour. The relatively low Faradaic efficiencies can be explained by various reasons: (1) some photocurrent produced from the tandem results from dye detachment from the TiO_2_ surface, which was visibly observed on the films post reaction; (2) some current may arise from slight degradation of the photocathodes; (3) quantifying H_2_ evolution *via* headspace analysis of the PEC cell does not account for any dissolved H_2_ in the electrolyte within the cell or any small leakages.

### Spectroelectrochemistry and transient absorption spectroscopy

**Fig. 6:**
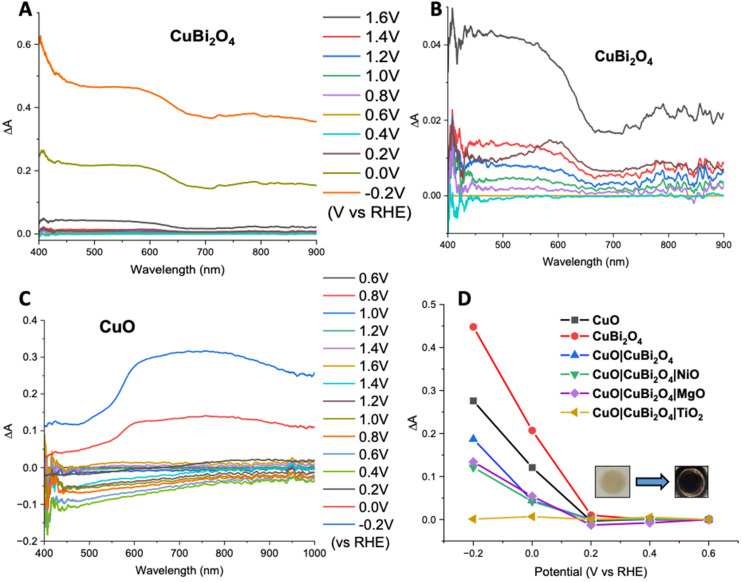
Spectroelectrochemical analysis. (A) Change in absorbance (Δ*A*) of CuBi_2_O_4_ at a series of applied potentials, and (B) expanded for the range between −0.2 and 1.0 V. (C) Δ*A* of a CuO film at a series of applied potentials. (D) Δ*A* at 600 nm *vs*. potential for all photocathode configurations. All spectroelectrochemical measurements were carried out at pH 7.2 in 0.2 M KCl, 0.01 M H_2_KPO_4_, and 0.01 M HK_2_PO_4_ (pH 7.2) with a 3.5 M Ag/AgCl reference electrode and a Pt wire as a counter electrode.

Spectroelectrochemistry^34^ and transient absorption spectroscopy (TAS)^35^ were used to gain a deeper understanding of charge dynamics within the semiconductors. Spectroelectrochemistry was employed using a combination of CV and UV-visible absorbance spectroscopy to study the redox chemistry at the semiconductor–electrolyte interface. Throughout the CV an absorption spectrum was recorded at various voltages for both CuO and CuBi_2_O_4_. Fig. 6A and B shows the difference in absorption spectra obtained at different applied voltages for CuBi_2_O_4_. Fig. 6B shows that upon the initial oxidative sweep (from 0.6 V to 1.6 V *vs*. RHE), the absorbance between 400–650 nm starts to increase steadily until 1.4 V. A larger increase in absorbance was displayed at 1.6 V. During the return sweep, all changes in absorbance were found to be reversible. Little change in absorbance was observed at 0.4 V. However, at 0.2 V an increase in absorbance was observed at 600 nm. This feature resembles the absorbance spectrum of metallic Cu. As the potential was decreased further to 0 V and −0.2 V, more significant increases in the absorbance were observed. The colour of the films also changed from brown to black. These changes in absorbance also correlate with the reduction peaks observed during CV experiments (Fig. S17†) and can be attributed to an increasing concentration of Cu^1+^ and Cu^0^ sites at 0.2 V and −0.1 V respectively. The reduction of Bi^3+^ sites forming metallic Bi is also thought to occur at −0.2 V, which also correlates with published values for these redox processes.^36^ These reduction peaks were also found to be reversible, according to both the CV and the absorption spectra.

A similar trend was observed for the CuO sample during the oxidative sweep, with the absorbance increasing upon the application of a positive potential (1.6 V). When the potential was then swept back to the to 0.4 V, a gradual decrease in the absorbance was observed. The intensity of this band appeared to decrease over time, independently of the applied voltage, and no redox peaks were observed in the relevant region on the CV. A reason could be that the spray pyrolyzed films of CuO displayed poor adhesion to the FTO substrates and the addition of the CuBi_2_O_4_ to the surface strengthened the assembly. After the experiment, a visible colour change could be observed on the CuO film, confirming that slight CuO detachment occurred during the positive sweep of the CV. For the unprotected electrodes, a plot of Δ*A* at 600 nm *vs.* applied potential showed that at potentials ≤0.2 V *vs*. RHE, a large increase in absorbance occurs. This is consistent with the degradation discussed above. However, upon addition of a protective material, this change in absorbance was significantly smaller. This supports the explanation that TiO_2_, MgO, and NiO effectively protect the electrode–electrolyte interface, possibly reducing the concentration of intra-band gap states. This effect was most prominent in the TiO_2_ sample, where no change in absorbance was observed, even at −0.2 V *vs*. RHE (Fig. 6D). The inset in Fig. 6D shows the photographs illustrating a visible colour change to the CuBi_2_O_4_ when the electrode was held at −0.2 V *vs*. RHE for 5 minutes.

**Fig. 7:**
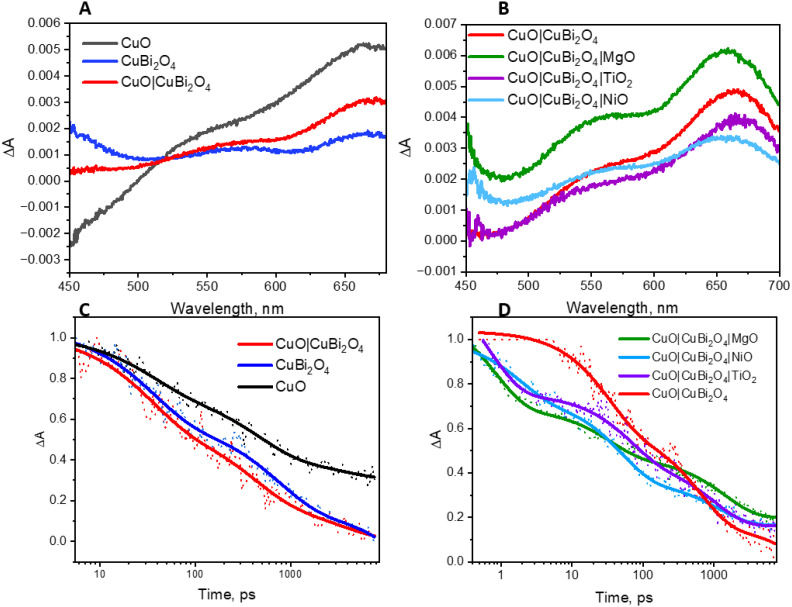
Transient absorption spectroscopy. (A) TAS of CuO, CuBi_2_O_4_, and the combined sample at a time delay of 10 ps. (B) TAS showing the effect of each protective layer in the presence of electrolyte at a time delay of 10 ps. (C) Decay of transient absorption for CuO, CuBi_2_O_4_, and the combined sample. (D) Decay of transient absorption, showing the effect of each protective layer in the presence of electrolyte. The excitation wavelength of the probe pulse was 400 nm for each measurement.

The charge-transfer dynamics of the excited semiconductors were investigated using TAS (Fig. 7). Broad transient absorption bands appeared following excitation of the CuO and CuBi_2_O_4_ films and the mixed samples, CuO|CuBi_2_O_4_ (Fig. 7A). The peaks observed at 560 nm and 660 nm for CuBi_2_O_4_ can be attributed to ligand-to-metal charge transfer transitions between Bi–O and Cu–O, respectively.^37^ The CuO films produced a distinctive TA spectrum, containing a bleach at 450 nm and a transient absorption band at 675 nm, consistent with a charge transfer transition. The spectra for the CuO|CuBi_2_O_4_ samples were similar to CuO, but there was a slight difference in shape of the charge transfer band. A transient absorption band at 600 nm decayed rapidly (mostly within 5 ns). The lifetime of the transient was shorter than those recorded for CuO or CuBi_2_O_4_ alone (Fig. 7). Fitting these bands to exponential decay functions gave lifetimes of 3.72 ± 0.5 ns and 1.49 ± 0.17 ns, respectively (Table S27†). For the CuO|CuBi_2_O_4_ film, the lifetime of the transient was 0.94 ± 0.038 ns, which was significantly shorter compared to CuO and CuBi_2_O_4_ alone. This finding is consistent with a charge transfer process across the heterojunction between the two metal oxides.

The impact of adding protective layers on the CuO|CuBi_2_O_4_ surface was also tested, both with dry films and with films coated with electrolyte solution. Fig. 7B shows the effect of the protective coatings on the TA spectra of CuO|CuBi_2_O_4_. Upon the addition of TiO_2_, a slight red shift was observed at *ca.* 650 nm. This is consistent with a difference in the electronic environment at the surface, possibly due to a lower concentration of high-valence states which are responsible for some charge-transfer transitions associated with the optical absorption bands. In contrast, adding NiO or MgO resulted in blue shifts of the transient absorption bands. For the MgO-coated sample, the feature at 550 nm was slightly enhanced relative to the 650 nm peak whereas the TiO_2_-coated sample had a similar spectral shape to the unprotected sample in that region. These observations could be a combination of various effects including a difference in the electric field, dielectric environment or passivation mechanism at the surface. The differences may be due to Ti^2+^ having a larger ionic radius than Cu^2+^, Ni^2+^ and Mg^2+^ (which have similar radii).

The decay of the transient absorption for each sample is shown in Fig. 7D. The initial decay within the first 100 ps was faster for the three samples containing protective layers. However, after 1000 ps, the decay was slightly slower for the protected samples compared to the unprotected sample. The initial decay may be associated with enhanced charge separation and extraction which competes with charge-trapping by surface states. We propose that the protective layers facilitated rapid initial decay by quickly removing charge carriers from the recombination sites. The slower decay after 1000 ps could be due to the prevention of surface recombination processes *via* intra-band gap states, prolonging the lifetime of remaining charge carriers. Any new or modified charge-trapping states resulting from the additional layers may also slow down the decay. The spatial charge separation that occurs from the cascade design could also be responsible for the slower decay. This corresponds with the observed PEC performance and the enhancement in photocurrent that was observed in the CuO|CuBi_2_O_4_ photocathodes (Fig. 3B).

The lifetimes are presented in Table S4.† For the TiO_2_-coated samples, the lifetime increased for both the wet and dry films, which may be caused by TiO_2_ passivating intra-band gap states at the surface and thus slowing the rate of decay *via* these to the ground state. A similar trend was observed with the NiO-coated electrode when dry. However, in the presence of electrolyte, the lifetime was similar to the unprotected sample (*ca.* 1.18 ± 0.15 ns). This suggests that the affect is suppressed when the NiO is exposed to electrolyte. Finally, a similar extension of the lifetime of the transient absorption for the MgO-protected samples to the TiO_2_ samples was observed for the electrodes immersed in electrolyte and a very slight increase in lifetime when dry. These results suggest that there is a passivating effect in both the MgO and TiO_2_-coated electrodes. It is possible that the slightly shorter lifetime when exposed to the electrolyte is related to the photocatalytic reaction.

## Conclusions

In this work, critical insights into the charge dynamics and stability of Cu-based photocathodes have been studied in depth. Our approach has highlighted the potential of Cu-based semiconductors as affordable, and potentially very efficient components in solar-driven PEC water-splitting applications. MgO, NiO, and TiO_2_ were utilized as a coatings which enhanced photocathode stability whilst facilitating efficient charge transfer. The results show that MgO was the most efficient of the three materials trialled. This presents new avenues for designing protective layers if a thin enough layer of material is used. This insight has advanced the fundamental understanding of charge recombination and protection strategies in Cu-based photocathodes and ‘sandwich’ type multi-layered structures. Our findings can lead to applying similar protective approaches across a wider range of PEC systems.

The in-depth chemico-physical, spectroscopic and electrochemical analysis of the CuO|CuBi_2_O_4_ photocathodes with TAS and spectroelectrochemistry provided a details of charge transfer, recombination dynamics, and stabilizing effects. Spectroelectrochemistry revealed that potentials <0.1 V *vs*. RHE result in an increase in optical density, which accompanied degradation of the semiconductors. This degradation was reduced upon the application of TiO_2_, MgO, or NiO. TAS showed effective charge transfer between CuO and CuBi_2_O_4_ with a quenched lifetime displayed for the CuO|CuBi_2_O_4_ heterojunction. Overall, slower kinetics were observed in the presence of protective layers, which was attributed to reduced surface-recombination.

By addressing the topical and longstanding challenge of photocorrosion of Cu-based photocathodes, this study underscores the potential of abundant and inexpensive materials, with a specific highlight on the novel usage of MgO.

On an applied level, this paper focusses on Pt and MoS_2_ as co-catalysts for HER with the highest performance produced from CuO|CuBi_2_O_4_|MgO|Pt (*ca.* −0.2 mA cm^−2^ at 0.4 V *vs*. RHE). Brief analysis of MoS_2_ as a viable alternative to Pt suggested that it was capable of enhancing the photocurrent of CuO|CuBi_2_O_4_|MgO on a similar scale to Pt. Future work could focus on the effect of MoS_2_ in long term experiments. All photoelectrodes were tested for PEC H_2_ evolution from neutral (pH 7.2) water at 0.4 V *vs*. RHE. Results for the CuO|CuBi_2_O_4_|MgO|Pt configuration showed that after 3 hours of reaction a high Faradaic efficiency was observed at 92.1% with *ca.* 1.81 ± 0.24 mmol cm^−2^ of H_2_ evolved. However, the formation of pinholes in the protective layer remains an issue for stability. This study successfully demonstrated unassisted tandem water splitting using the CuO|CuBi_2_O_4_|MgO|Pt photocathode which was paired with a dye-sensitized TiO_2_ photoanode. Bias-free chronoamperometry was performed for 60 minutes of a TiO_2_|LEG4||Pt||CuO|CuBi_2_O_4_|MgO|Pt tandem producing a photocurrent of 75 μA cm^−2^ with 93% of the photocurrent remaining after 60 minutes. After 1 hour, 378 ± 25 nmol cm^−2^ of H_2_ had been evolved at aFaradaic efficiency of 58.3 ± 3.7%. The achievement of overall water splitting without external bias using metal oxide and molecular light-absorbers highlights the potential for efficient and durable solar H_2_. Continued fundamental study and application of these technologies will bring renewable H_2_ production closer to practical implementation.

Future work should focus on the development of key strategies to deposit, thin, uniform, and pinhole-free protective coatings to maintain electrode performance. This will allow the production photoelectrodes with longer lifetimes for excited charge, to accelerate the implementation of PEC systems on an industrial scale and aiding in the global production of green H_2_. Other applications should also be investigated, including utilizing these photocathodes for CO_2_ utilization in microbial photoelectrosynthesis cells to produce liquid chemicals from sunlight.^38^

In summary, the findings presented in this paper offer a robust basis for future exploration, highlighting that the materials explored could revolutionize solar-driven PEC H_2_ production. The study has enhanced both practical and fundamental understanding with advances made in protective layer design, stability challenges, and unravelling charge transfer dynamics. The findings in this study should serve as a stepping stone for future investigations into Cu-based metal oxide photoelectrodes, promoting an economical method for solar fuel production.

## Methods

### Materials

All chemicals were purchased from Sigma-Aldrich and were used without further purification unless stated otherwise.

### PEC characterization

For single photoelectrode systems, photoelectrochemical measurements were carried out in a 3-electrode photoelectrochemical cell consisting of a working electrode (photoanode/photocathode), an Ag/AgCl reference electrode, and a Pt wire as a counter electrode. The working area was 0.79 cm^2^ which was masked using an O-ring within a custom-built PEC glass cell. The electrolyte used in each experiment (unless stated otherwise) was 0.2 M KCl, 0.01 M H_2_KPO_4_, and 0.01 M HK_2_PO_4_ (pH 7.2). A 300 W Xe lamp calibrated using to 1 sun (filtered with an AM1.5G filter) was used for photoelectrochemical experiments. The lamp was calibrated using a standardized silicon photodiode.

### Product analysis *via* gas chromatography

The headspace volume was maintained at 7 mL for all experiments. H_2_ evolution was quantified *via* direct injection into a Simadzu gas chromatograph using a 2.5 mL Hamilton gas syringe with a constant from rate of Ar carrier gas of 1 ml min^−1^ and a thermal conductivity detector (TCD) was used, operated at 250 °C. 1 mL of gas was measured from the headspace of the PEC reactor. Charge passed was calculated by integrating the each chronoamperometry and this was used to calculate the Faradaic efficiency. The process was repeated three times to interpret error values. The Faradaic efficiency was calculated based on the following equation:
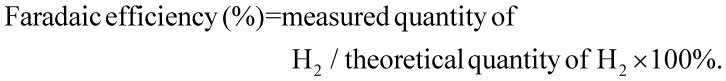


### Structural and morphological characterization

Film morphology was studied using a Tescan Mira3 scanning electron microscope (SEM). Energy-dispersive X-ray analysis (EDS) was carried out using the SEM and was analysed using an Oxford instruments EDS analyser. Film structure was determined by X-ray diffraction (XRD) of the thin films on FTO glass using a Rigaku SmartLab X-ray diffractometer. The absorption spectra of films was recorded using an ocean optics fibre optic setup connected to LS-1 light source and USB2000 detector. XPS analysis was performed using a Thermo Scientific K-alpha X-ray Photoelectron Spectrometer™ (Thermo Scientific, East Grinstead, UK). Survey spectra, capturing broad energy ranges and multiple elements, were utilized to obtain elemental data from scans ranging from −5.0 eV to 1350.0 eV. All scans were calibrated to the carbon 1s peak at a binding energy of 284.8 eV. Spectra acquisition was achieved using a monochromatic Al Kα X-ray source with an output energy of 1486.6 eV. Spectral analysis was executed with CasaXPS software (CasaXPS Ltd).

### Absorption characterization

UV-visible absorption and spectroelectrochemical measurements were conducted using an Ocean Optics USB 2000+.

### Electrochemical characterization

Electrochemical characterization measurements were conducted using a PalmSens EmStat3 Blue potentiostat to apply bias and record the current throughout the experiments with Ag/AgCl reference electrodes used for all experiments.

### CuBi_2_O_4_ synthesis

Synthesis of CuBi_2_O_4_ was carried out in an automated co-precipitation reactor. HPLC pumps were used to pump the Cu^2+^ and Bi^3+^ precursor solutions at controlled flow rates through a T-piece junction to control the stoichiometry of the metal hydroxide nanoparticles. The Cu^2+^ and Bi^3+^ salts were then slowly pumped into the reactor in a ratio of 1 : 2, respectively. The OH^−^ results in hydrolysis and dehydration of the Cu and Bi salts, precipitating a green solid, CuBi_2_(OH)_4_. The precipitated product was washed with deionised water, re-centrifuged multiple times, dried and sintered in air at 650 °C to complete oxidation from CuBi_2_(OH)_4_ to CuBi_2_O_4_. CuBi_2_O_4_ powder was then ground in a Retsch PM100 plantary ball mill for 12 hours to decrease particle size and increase uniformity of particle size. The resulting suspension (in ethanol) was filtered through a wire gauze sieve with 20 μm pores to remove any larger agglomerates and washed with further ethanol. The paste recipe consisted of a mixture of terpineol (5 g), and ethyl cellulose (6 g) along with 2 g of the CuBi_2_O_4_. Excess ethanol was then evaporated off under reduced pressure until the desired paste thickness was achieved.

### Cleaning FTO substrates

TEC 15 fluorine doped tin oxide (FTO) glass (Pilkington NSG) was cut into 2 cm × 2 cm substrates. FTO substrates were then cleaned *via* a three-step ultrasonication process; in 10% Hellmanex solution (15 minutes), deionised water (15 minutes), and in ethanol (15 minutes). The substrates were then dried using compressed air and cleaned under a UV ozone cleaner for 15 minutes to remove any remaining organics from the surface.

### Photocathode fabrication

For spray pyrolyzed samples, a solution of 0.04 M CuNO_3_·*x*H_2_O was sprayed onto the substrates at a height of 18 cm at 450 °C, a speed of 10 cm s^−1^, and a total of 20 spray cycles leaving 1 minute between each spray cycle. CuBi_2_O_4_ paste was doctor bladed onto clean FTO substrates and CuO coated FTO substrates using Scotch tape (Scotch Magic Tape™ 19 mm × 33 m invisible – typically ∼50 mm thick) as a spacer. The paste was annealed to the FTO substrates at 450 °C for 30 minutes with a 30-minute ramp time. Protecting layers were deposited from a solution of titanium isopropoxide (TTIP) in isopropanol in a ratio of 1 : 50 for TiO_2_ deposition, 0.5 M nickel acetate tetrahydrate (Ni(CH_3_COO^−^)_2_·4H_2_O) was made up in 2 methoxyethanol for NiO deposition, and a 1 : 50 (w/v) magnesium acetate (Mg(CH_3_COO^−^)_2_·4H_2_O) : 50% ethanol solution for MgO deposition. Each solution was sonicated for 15 minutes before use. The solutions were drop-cast onto the electrode surface and allowed to rest for >30 seconds before spin coating at 3000 rpm for 30 seconds. The electrodes were heated to 450 °C for 30 minutes with a 30-minute ramp time to ensure adhesion between the Cu-layer and each protective layer.

### Deposition of Pt/MoS_2_ HER catalysts

3 mL cm^−2^ of a 4.8 mM platinum(ii) bis(acetylacetonate) (Pt(acac)_2_) solution in acetone was drop cast onto the surface of the photoelectrodes after being sonicated for 30 minutes. The volume of solution was varied to optimise the HER conditions. The electrodes were then annealed at 450 °C for 15 minutes with a 15-minute ramp time to form colloidal Pt particles on the surface of the electrodes. A similar procedure was followed using a 4.8 mM suspension of MoS_2_ nanoparticles in IPA with an average particle size of 90 nm, purchased from Sigma Aldrich (1317-33-5). The suspension was sonicated for 15 minutes before deposition of 7.2 nmol cm^−2^ being deposited onto the electrode surface. Particles were then annealed to the photocathode surface using gentle heating to 200 °C for 30 minutes.

### Photoanode fabrication

Mesoporous TiO_2_ (CAS: 791547) was deposited onto clean FTO substrates *via* blade coating using Scotch Magic Tape™ to mask the edges. The films were then annealed at 500 °C for 30 minutes. The samples were allowed to cool gradually to room temperature. For samples with Pt co-catalyst present, 7.2 nmol cm^−2^ was deposited onto the TiO_2_ films and the electrodes were then annealed at 450 °C for 15 minutes with a 15-minute ramp time to form colloidal Pt particles on the surface. Dye-sensitization was then carried out by submerging the films in a LEG4 dye bath (approx. 3 mM dye in dry acetonitrile) for 12–15 hours and washed with acetonitrile and dried in air before any photoelectrochemical reaction.

### Tandem cell configuration

For dual photoelectrode systems, photoelectrochemical measurements were carried out in a 2-electrode photoelectrochemical cell consisting of a working electrode of CuO|CuBi_2_O_4_|MgO|Pt photocathode and a TiO_2_|LEG4|Pt as a counter electrode. The working area was 0.79 cm^2^ which was masked using an O-ring within a custom-built PEC glass cell. Illumination was carried out through illuminating the photoanode at the front with the photocathode being placed directly behind. The electrolyte used in each experiment (unless stated otherwise) was 0.2 M KCl, 0.01 M H_2_KPO_4_, and 0.01 M HK_2_PO_4_ (pH 7.2). A 300 W Xe lamp calibrated using to 1 sun (filtered with an AM1.5G filter) was used for photoelectrochemical experiments. The lamp was calibrated using a standardized silicon photodiode.

### Spectroelectrochemistry

Spectroelectrochemical experiments were performed on films of the various semiconductor configurations on FTO suspended in an neural buffered solution (0.2 M KCl, 0.01 M H_2_KPO_4_, and 0.01 M HK_2_PO_4_ (pH 7.2)). UV-Vis spectroscopy was carried out using an ocean optics fibre optic setup connected to LS-1 light source and USB2000 detector. Cyclic voltammetry (CV) was performed on the samples at a scan rate of 1 mV s^−1^ and absorbance measurements were acquired every 100 mV. CV was performed using a PalmSens EmStat3 Blue potentiostat.

### TAS

Experiments were conducted using a Helios spectrometer from Ultrafast Systems, with a Solstice Ace laser (Spectra Physics) generating pump and probe beams. The laser produced pulses of 800 nm light with a duration of 100 fs at 1 kHz which was passed through a sapphire crystal to form a white light continuum. The pump light was tuned to the desired wavelength using an optical parametric amplifier (OPA) model Apollo-T from Ultrafast Systems. Spectra were recorded using a fiber-coupled CCD array. Excitation wavelength of the pump pulse was 400 nm for each measurement. The films containing CuO were synthesized following the spray pyrolysis technique with only 10 spray cycles to decrease the optical density and allow absorbance to be measured. CuBi_2_O_4_ paste was diluted by 50% in ethanol for the same reason. Samples with protective layers were carried out following the same spin-coating method as above. Measurements were conducted on films on FTO glass substrates. CuO|CuBi_2_O_4_ and all protected samples were tested dry and with electrolyte (0.2 M KCl, 0.01 M H_2_KPO_4_, and 0.01 M HK_2_PO_4_ (pH 7.2)) dropped onto the surface of the electrodes and topped with a microscope slide. Global analysis was performed using Surface Xplorer. Kinetic fitting was modelled in Origin.

## Author contributions

C. Burns, E. A. Gibson, and S. Kalathil conceptualized the project. C. Burns carried out the experiments and wrote the manuscript. S. L. Stephens built and coded the co-precipitation reactor in which CuBi_2_O_4_ nanoparticles were synthesized and aided in the initial synthesis. M. Rishan carried out the XRD analysis of the photocathodes. O. Woodford and C. Burns conducted TAS measurements. E. A. Gibson, L. Fuller, and S. Kalathil supervised the project, edited the manuscript, and acquired the funding for the project. All authors have given approval to the final version of the manuscript.

## Conflicts of interest

The authors declare no competing financial interests.

## Supplementary Material

DT-054-D4DT02738H-s001

## Data Availability

The data supporting this article can be found within the manuscript and the ESI.† Original data are available at data.ncl.ac.uk.
