# Supplementary material for: Interfacing CuO, CuBi2O4, and protective metal oxide layers to boost solar-driven photoelectrochemical hydrogen evolution
Source: Dalton Trans. 2024 Dec 4;54(3):920–33. doi: 10.1039/d4dt02738h (PMC11639663; doi:10.1039/d4dt02738h)
Supplement: DT-054-D4DT02738H-s001 [file DT-054-D4DT02738H-s001.pdf]

Supplementary Information (SI) for Dalton Transactions.
This journal is © The Royal Society of Chemistry 2024

Electronic supporting information for 'Interfacing CuO, $CuBi_2O_4$, and protective metal oxide layers to boost solar-driven photoelectrochemical hydrogen evolution.'.

Cathal Burns[a,b], Owen Woodford[b], Susanna L Stephens[b], Muhammed Rishan[a,b], Linsey Fuller[c], Shafeer Kalathil[a], Elizabeth A Gibson[b]**

- **a)** *Hub for Biotechnology in the Built Environment, Faculty of Health and Life Sciences, Department of Applied Sciences, Northumbria University, Newcastle, NE1 8ST, United Kingdom*
- **b)** *Energy Materials Laboratory, Chemistry, School of Natural and Environmental Science, Newcastle University, Newcastle upon Tyne, NE1 7RU, UK*
- **c)** *Procter & Gamble Innovation Centre, Whitley Road, Newcastle upon Tyne, NE12 9BZ, United Kingdom*

Table of Contents

***Additional Characterization of $CuBi_2O_4$*......2**

X-ray Diffraction......2

***Additional Characterization of $CuO|CuBi_2O_4$*......2**

X-ray Photoelectron Spectroscopy......2

Absorbance, Reflectance and Tauc Plots......4

SEM and EDS......6

***SEM*......8**

***Electrochemistry*......11**

***Gas Chromatography*......12**

***Post-PEC Characterization*......12**

***Additional Tandem Characterization*......14**

***Transient Absorption Spectroscopy Data*......15**

***Literature Comparison*......15**

***References*......17**

Additional Characterization of $CuBi_2O_4$

X-ray Diffraction

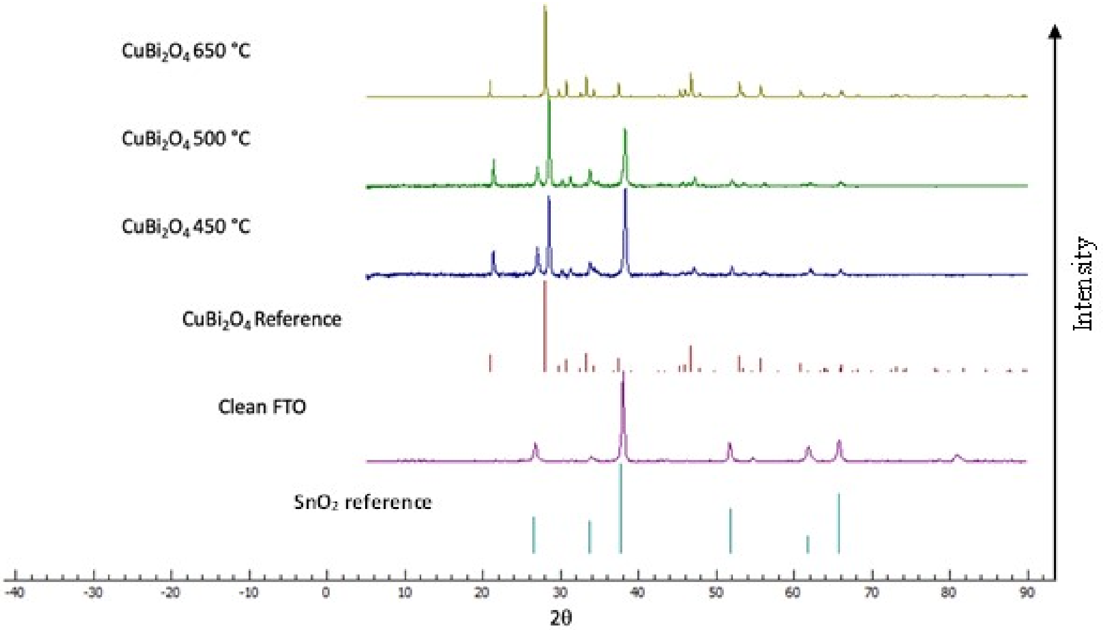

Figure S1 – X-ray diffractograms of $CuBi_2O_4$ at 450 °C, 500 °C, and 650 °C. FTO, SnO_2 reference diffractograms and a $CuBi_2O_4$ reference diffractogram from the literature (kusachiite, PDF no.00-042-0334).

Additional Characterization of CuO|$CuBi_2O_4$

X-ray Photoelectron Spectroscopy

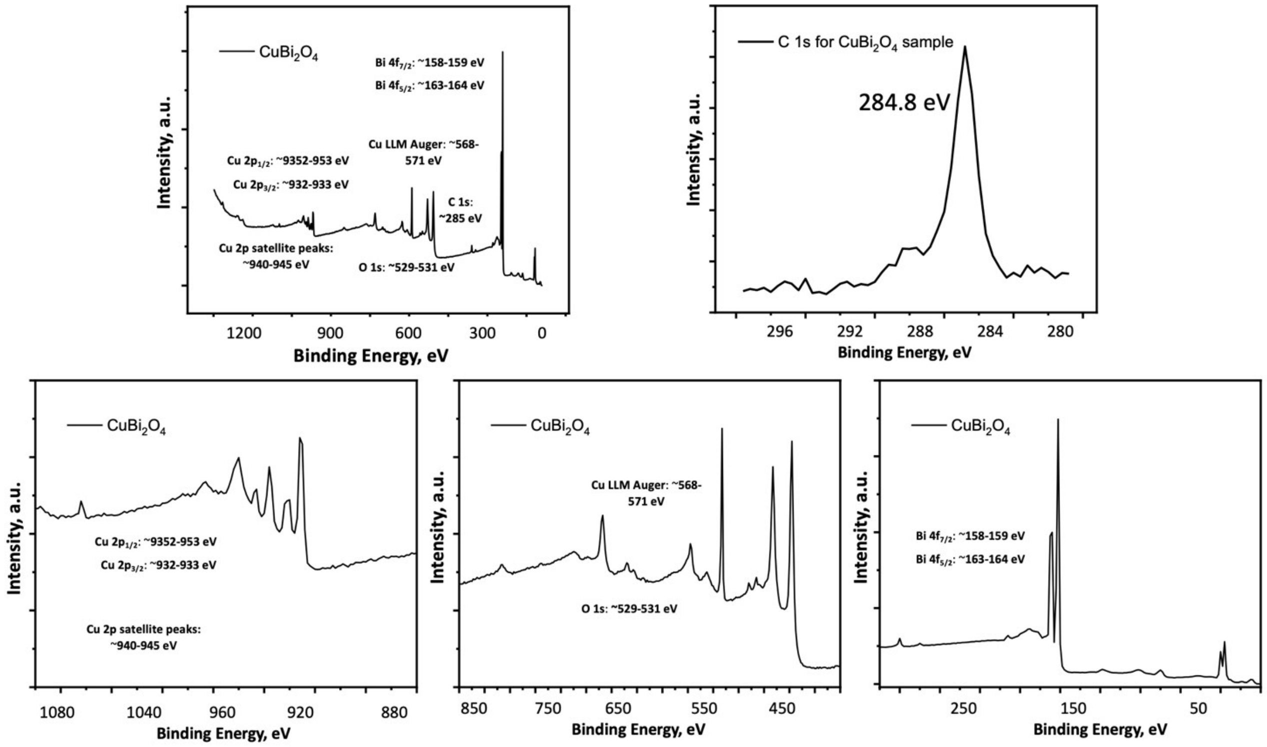

Figure S2 – **a-e)** XPS survey scans of $CuBi_2O_4$ showing the Cu 2p, Cu LLM Auger peaks, O 1s, C1s and Bi 4f peaks.

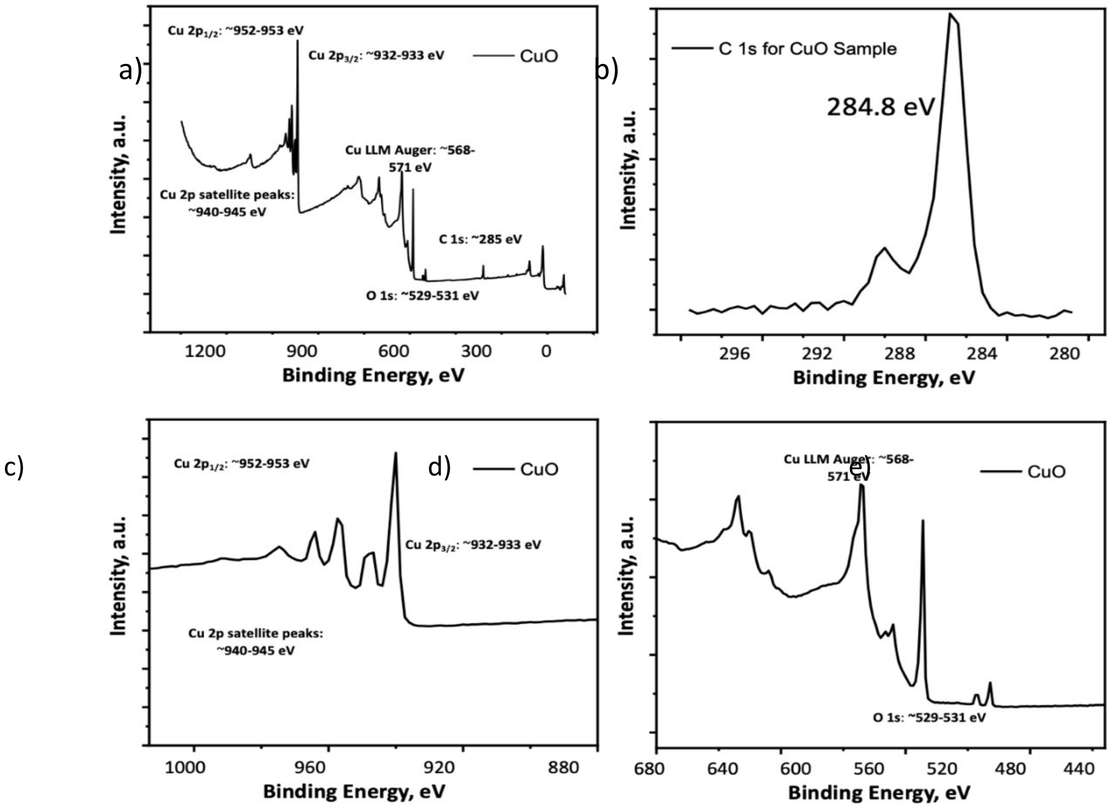

Figure S3 – **a-d)** XPS survey scans of CuO (bottom) showing Cu 2p, Cu LLM Auger peaks, O 1s, and C 1s peaks.

Survey Scans

a)

b)

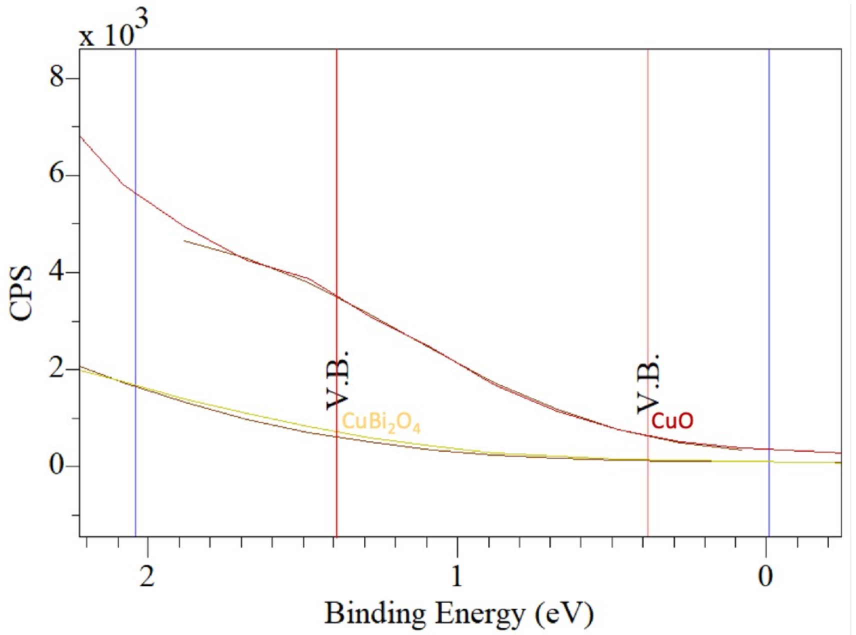

Figure S4 – Valence band X-ray photoelectron plot of CuO (red) and $CuBi_2O_4$ (yellow). The red vertical lines indicate the valence band positions (V.B.) for each material.

Valence Band Calculation

Material	Binding energies vs Fermi level (eV)
CuO	0.38
$CuBi_2O_4$	1.40

The valence band (VB) binding energy relative to the Fermi level ($E_{VB,F)}$ is defined as the difference between the VB maximum and the Fermi level. The Fermi level is defined as the highest energy an electron can have at absolute zero. In a semiconductor, the Fermi level lies between the VB and conduction band (CB). The Fermi level lies close to the VB in p-type

Table S1 – Calculated valence bands of CuO and $CuBi_2O_4$.

To calculate the VB energy vs Vacuum: $E_{VB,Vac} = E_{VB,F} - F$

F = Work function of a material

F-CuO = ~4.7-5.3 eV F-$CuBi_2O_4$ = ~4.7-5.3 eV

Valence band vs Vacuum:

$CuBi_2O_4$ = ~ 5.9 eV CuO = ~4.88 eV

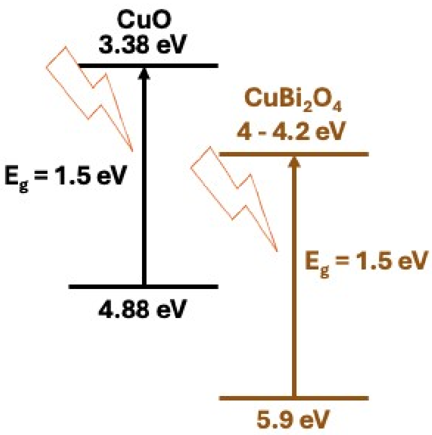

Absorbance, Reflectance and Tauc Plots

Figure S5 – Calculated band structure of the CuO|$CuBi_2O_4$ heterojunction.

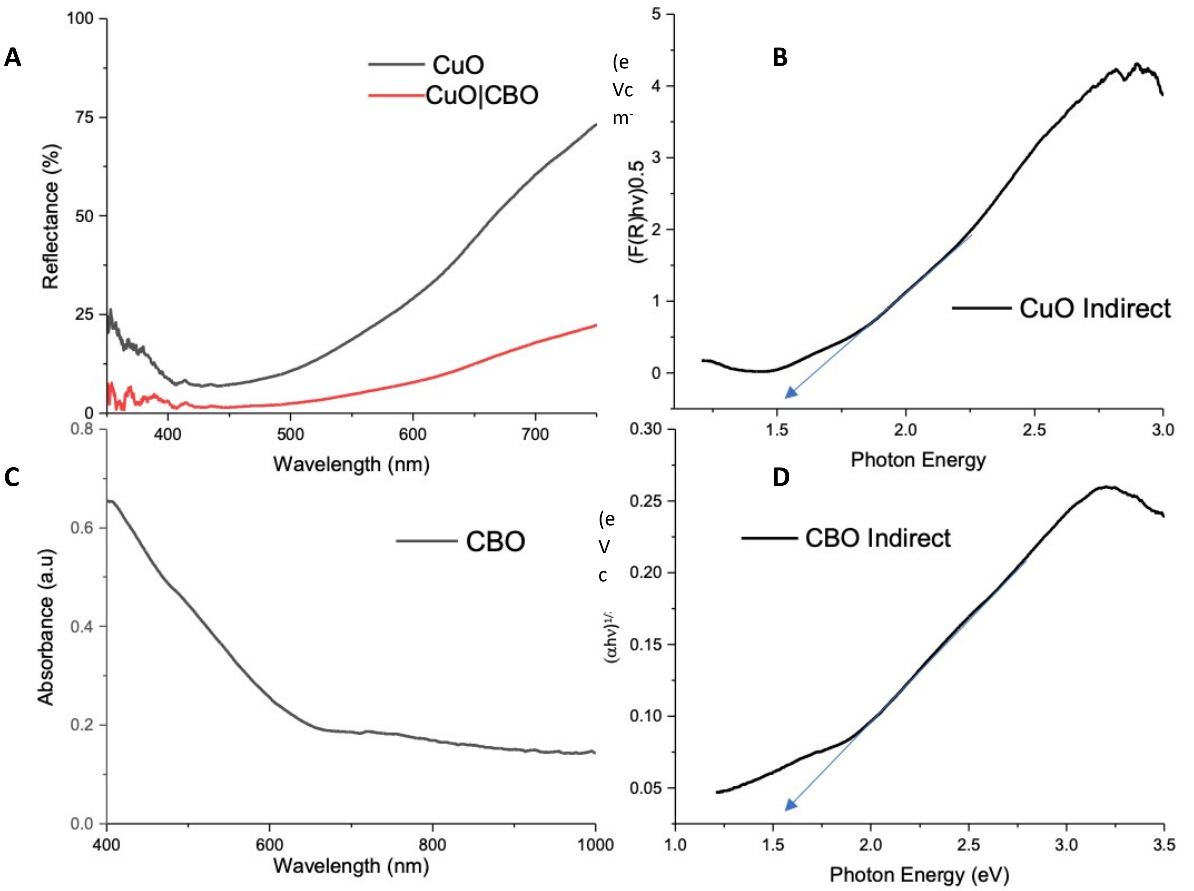

Figure S6 – **A)** Reflectance spectrum of CuO, and CuO|$CuBi_2O_4$. **B)** Tauc plot of CuO assuming an indirect bandgap. **C)** Absorbance spectrum of a $CuBi_2O_4$ film. **D)** Tauc plot for $CuBi_2O_4$ assuming an indirect bandgap.

SEM and EDS

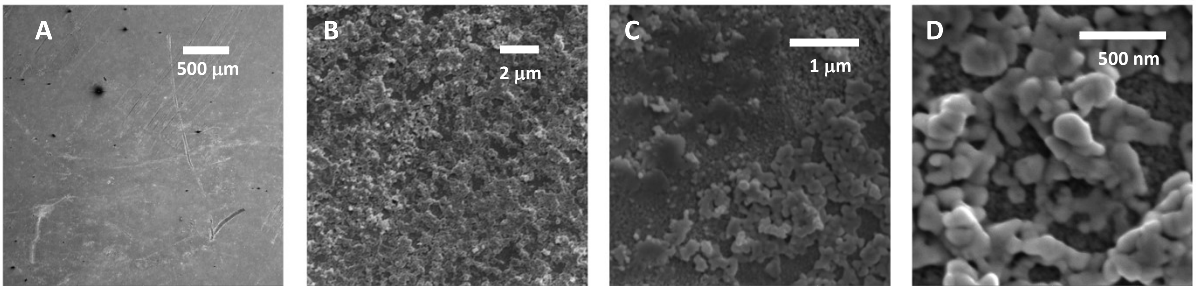

Figure S7 - SEM images of the CuO|$CuBi_2O_4$ electrodes. **A)** View field = 2.99 mm, magnification 118 x, and working distance (WD) = 11.64 mm. **B)** View field = 14.2 μm, magnification 24.8 kx, and working distance (WD) = 10.63 mm. **C)** View field = 4.15 μm, magnification 84.7 kx, and working distance (WD) = 10.64 mm. **D)** View field = 1.64 μm, magnification 215 kx, and working distance (WD) = 10.62 mm. (In-beam secondary electron detector with high voltage (HV)= 5.0keV for all images.)

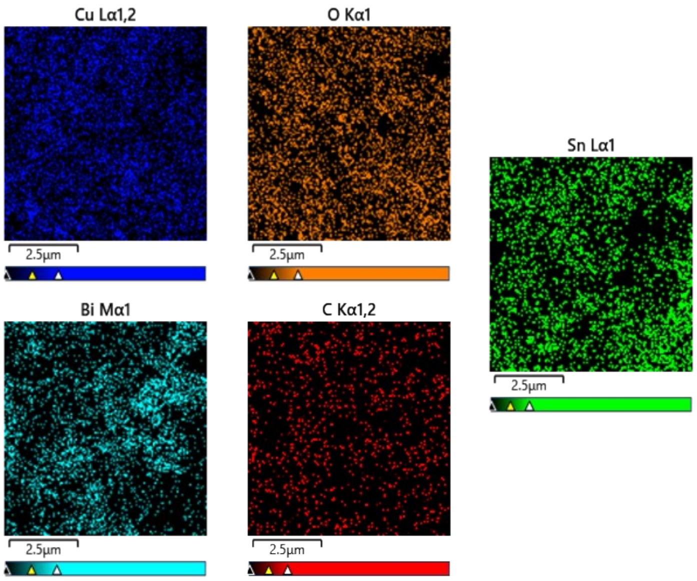

Figure S8 – SEM-EDS of FTO|CuO|$CuBi_2O_4$. The results show elemental mapping showing that Cu, Bi, O, C and Sn (from the FTO glass) can be found at the surface of the sample.

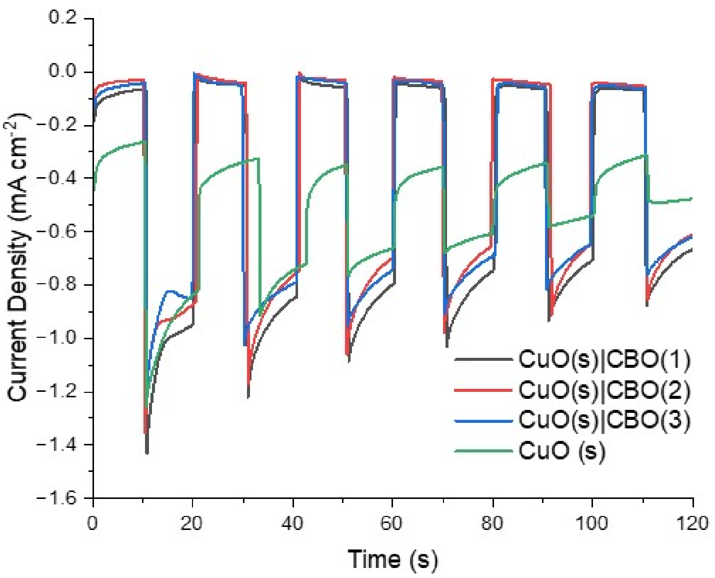

Figure S9 – Chopped-light chronoamperometry of CuO and CuO|$CuBi_2O_4$ with 1-3 layers of $CuBi_2O_4$ at 0.4 V vs RHE.

SEM

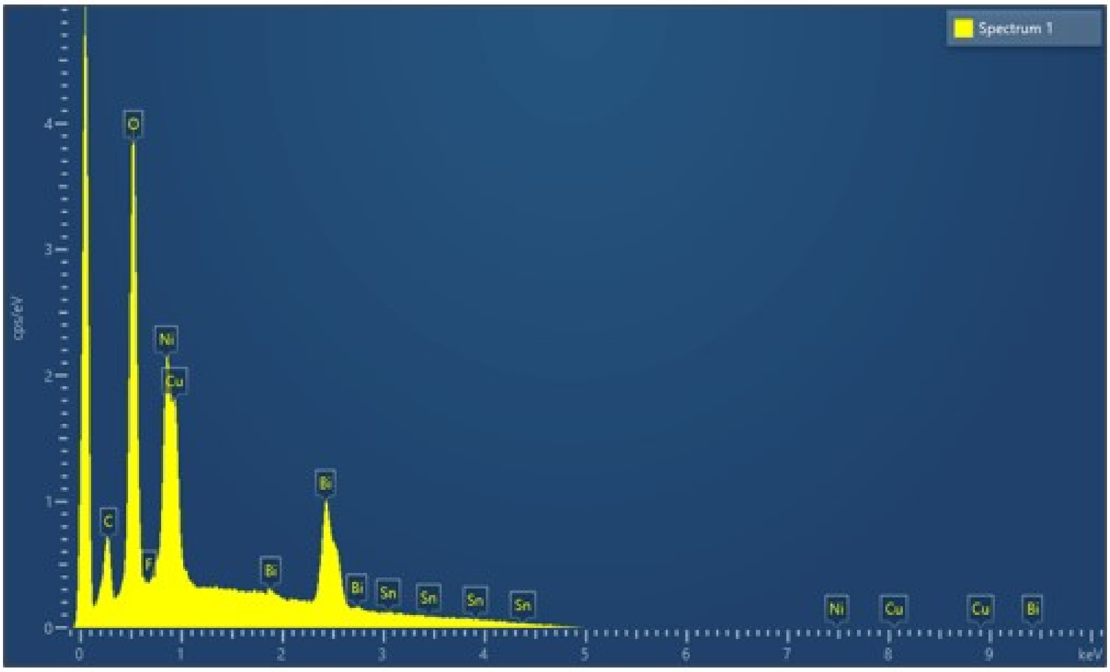

Element	Signal	Weight %	σ
Bi		53.57	0.63
Ni		17.53	0.38
O		15.17	0.25
Cu		11.72	0.30
C		1.75	0.09
F		0.26	0.13
Sn		0.00	1.97

Figure S10 – SEM-EDS data of FTO|CuO|$CuBi_2O_4$|NiO.

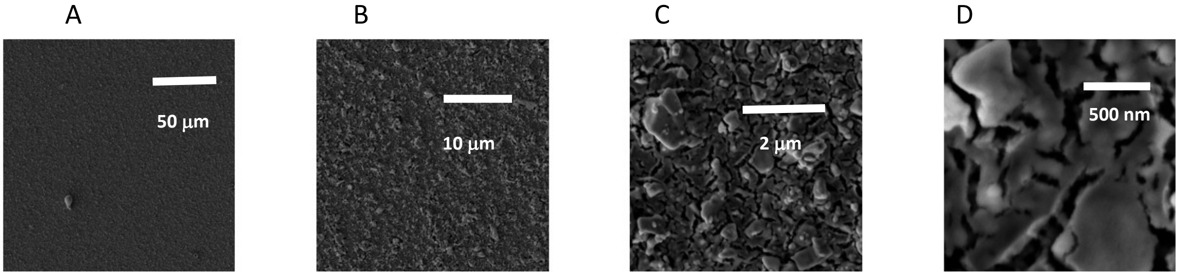

Figure S11 - SEM images of the CuO|$CuBi_2O_4$|NiO electrodes. A) View field = 192 μm, magnification 915 x, and working distance (WD) = 12.33 mm. B). View field = 32 μm, magnification 5.51 kx, and working distance (WD) = 12.36 mm. C) View field = 6.51 μm, magnification 27.0 kx, and working distance (WD) = 12.36 mm. D) View field = 1.62 μm, magnification 109 kx, and working distance (WD) = 12.36 mm. (Secondary electron detector with high voltage (HV)= 5.0keV for all images.)

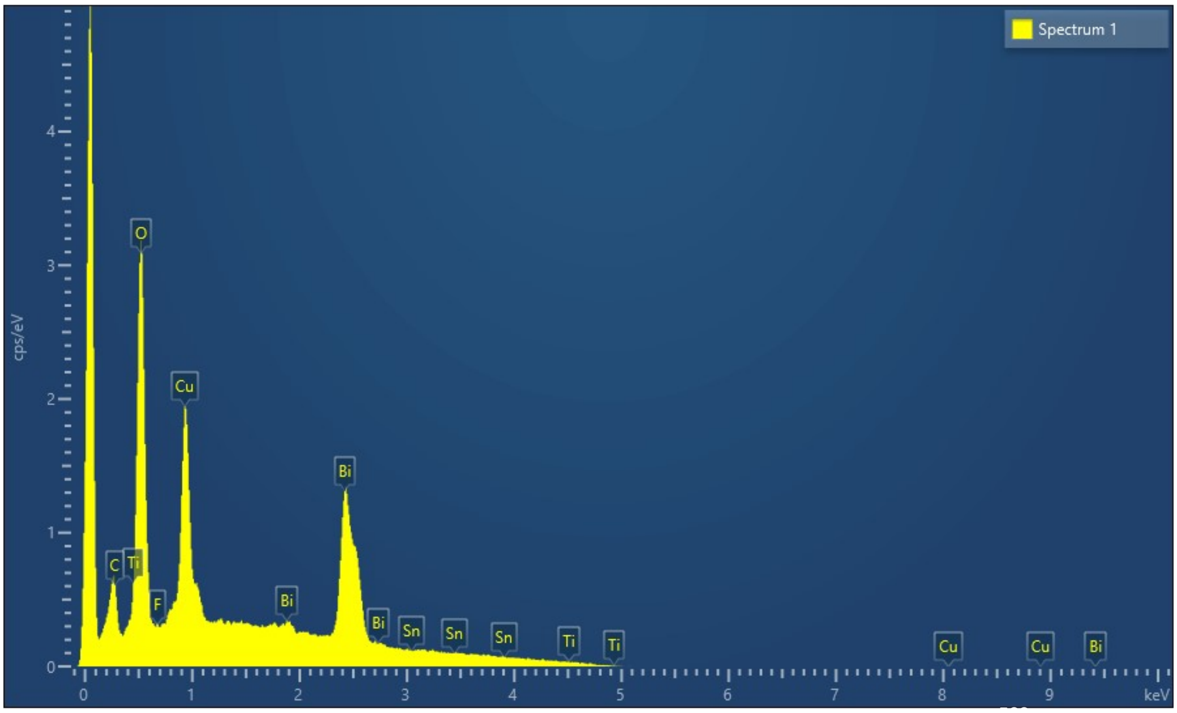

Element Signal	Weight %	σ
Bi	69.96	1.41
Cu	13.98	0.38
O	12.27	0.32
C	1.29	0.09
Sn	1.25	1.89
Na	1.25	0.10
F	0.00	0.11
Ti	0.00	2.90

Figure S12 - SEM-EDS data of FTO|CuO|$CuBi_2O_4$|TiO_2.

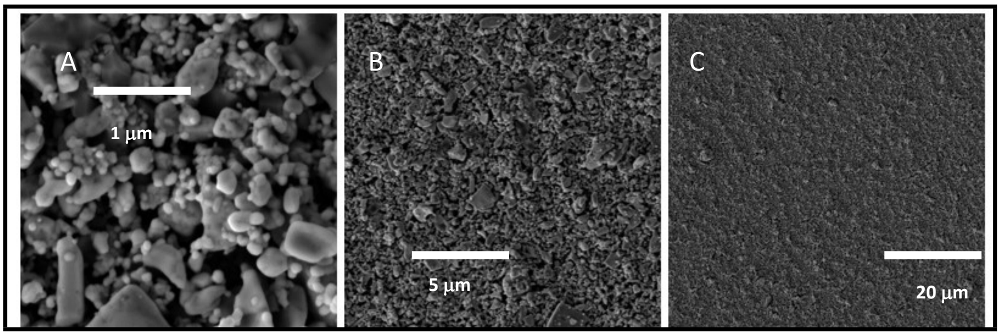

Figure S13 – SEM images of CuO|$CuBi_2O_4$|TiO_2. A) View field = 3.21 µm, magnification 73.2 kx, and working distance (WD) = 12.16 mm. B). View field = 15.7 µm, magnification 14.9 kx, and working distance (WD) = 12.17 mm. C) View field = 64.8 µm, magnification 2.72 kx, and working distance (WD) = 12.17 mm. (Secondary electron detector with high voltage (HV)= 5.0keV for all images.)

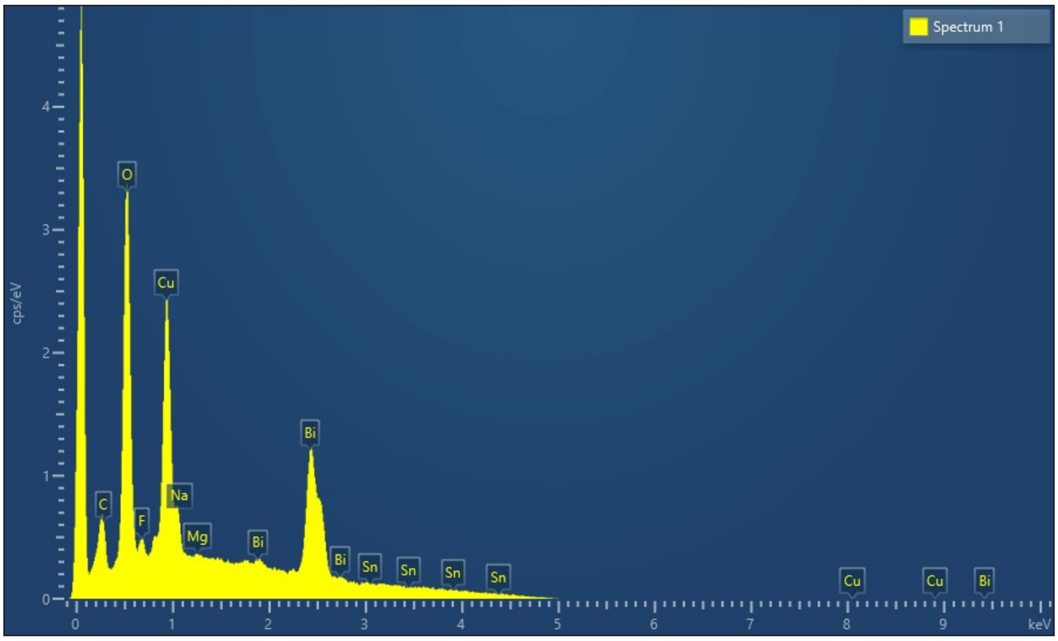

Element Signal	Weight %	σ
Bi	64.75	0.51
Cu	17.99	0.32
O	13.13	0.22
Na	1.75	0.11
C	1.27	0.09
F	1.00	0.12
Mg	0.11	0.07
Sn	0.00	1.94

Figure S14 – SEM-EDS data of CuO|$CuBi_2O_4$|MgO.

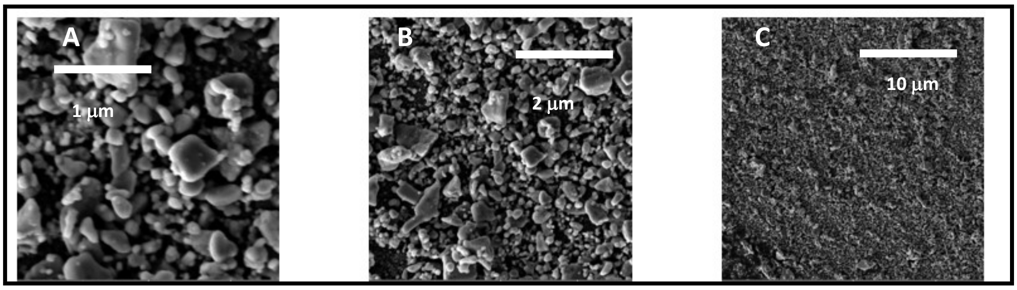

Figure S15 - SEM images of CuO|$CuBi_2O_4$|MgO. A) View field = 3.19 µm, magnification 73.6 kx, and working distance (WD) = 12.69 mm. B). View field = 6.86 µm, magnification 34.2 kx, and working distance (WD) = 12.7 mm. C) View field = 31.7 µm, magnification 7.4 kx, and working distance (WD) = 12.71 mm. (Secondary electron detector with high voltage (HV)= 5.0 keV for all images.)

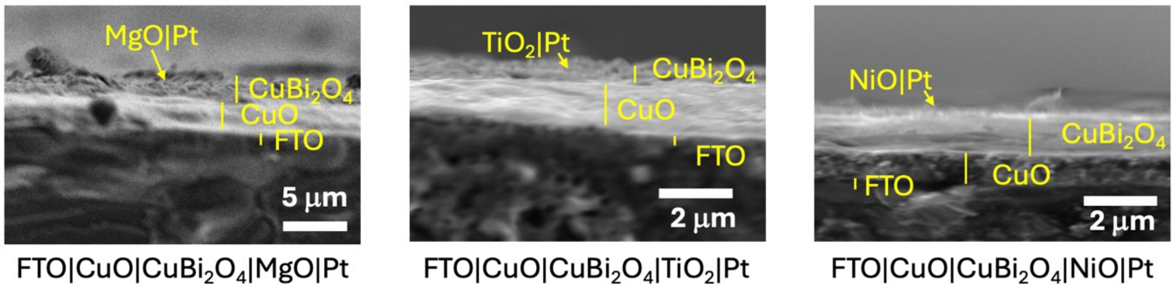

Figure S16 – Cross sectional SEM images of A) CuO|$CuBi_2O_4$|NiO|Pt, B) CuO|$CuBi_2O_4$|TiO_2|Pt, and C) CuO|$CuBi_2O_4$|MgO|Pt. A secondary electron detector with high voltage (HV)= 5.0 keV for all images.

Electrochemistry

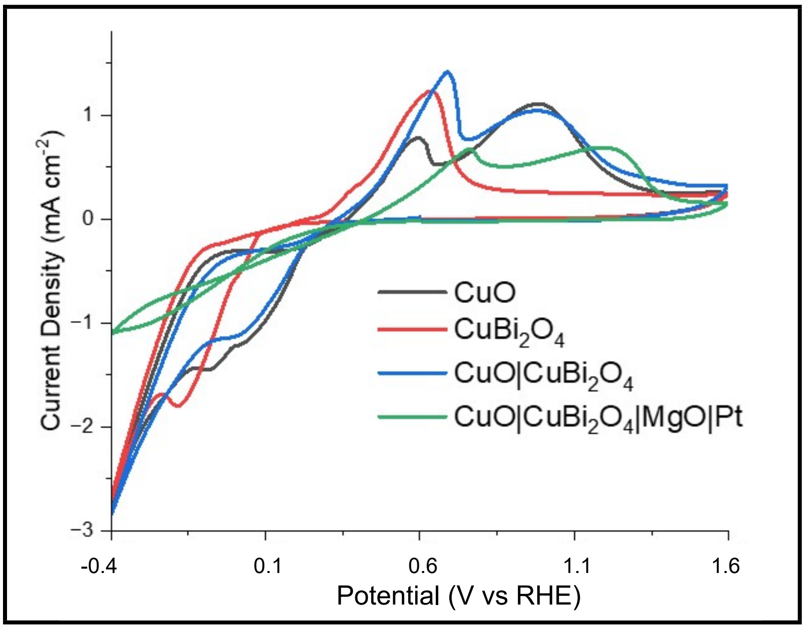

Figure S17 – Cyclic voltammetry of CuO, $CuBi_2O_4$, CuO|$CuBi_2O_4$, and CuO|$CuBi_2O_4$|MgO|Pt. Scan rate = 10 mV s^{-1}.

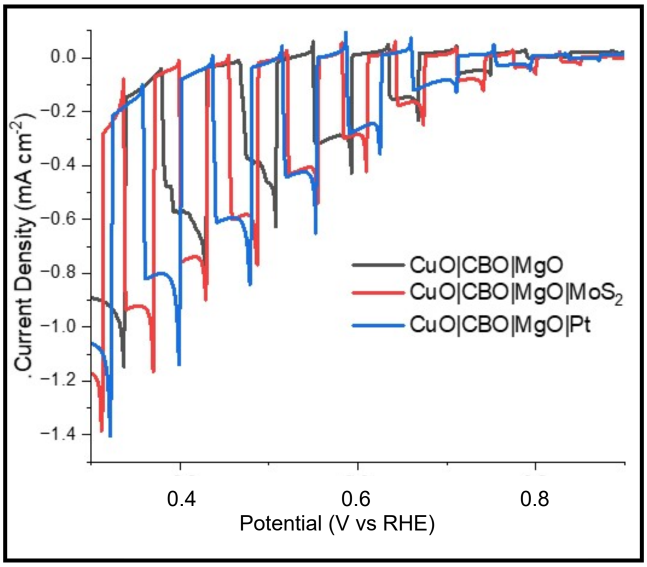

Figure S18 - Chopped Light LSV's comparing the effects of Pt and MoS_2 HER co-catalysts on CuO|$CuBi_2O_4$|MgO.

Gas Chromatography

Reaction Time (mins)	Applied V vs Ag/AgCl	pH	Charge passed	Electrons passed (mol)	Headspace volume (L)	H_2 peak	H_2 ppm	[H_2] / mol	Faradaic efficiency (%)
60	-0.2	7.2	2.41x10^{-1}	1.25 x10^{-6}	0.02	2461	357	1.18 x10^{-6}	95%
120	-0.2	7.2	5.00 x10^{-1}	2.59 x10^{-6}	0.02	4654	686	2.27 x10^{-6}	88%
180	-0.2	7.2	7.60 x10^{-1}	3.94 x10^{-6}	0.02	7405	1097	3.63 x10^{-6}	92%

Table S2 – Table showing the faradaic efficiency calculations of the CuO|$CuBi_2O_4$|MgO|Pt PEC cell at -0.2V vs Ag/AgCl with Pt wire as counter electrode. Experiments were carried out in a custom-built glass cell without an ion exchange membrane separating the counter and working electrodes.

ppm	mg m^{-3}	g/m^{-3}	mol m^{-3}	moldm^{-3}	mol
357	29.27	0.029	0.06	5.90 x10^{-5}	1.18 x10^{-6}
686	56.25	0.056	0.11	11.34 x10^{-5}	2.27 x10^{-6}
1097	89.95	0.090	0.18	18.13 x10^{-5}	3.63 x10^{-6}

Table S3 – Table showing ppm to moles conversion.

Post-PEC Characterization

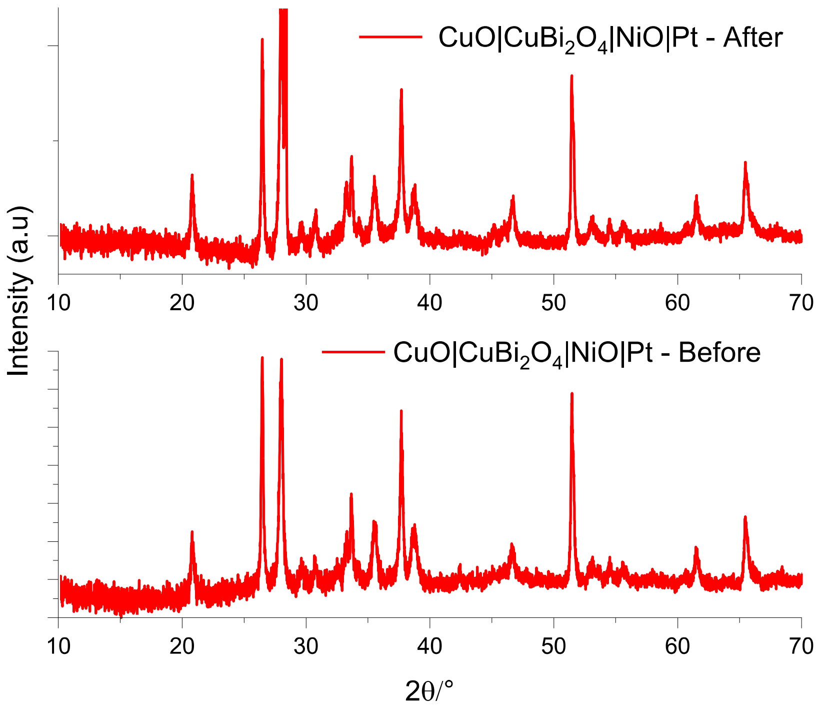

Figure S19 – X-ray diffractogram of CuO|$CuBi_2O_4$|NiO|Pt before and after 3 hours of PEC at 0.4V vs RHE and 1 sun illumination (300W Xe lamp).

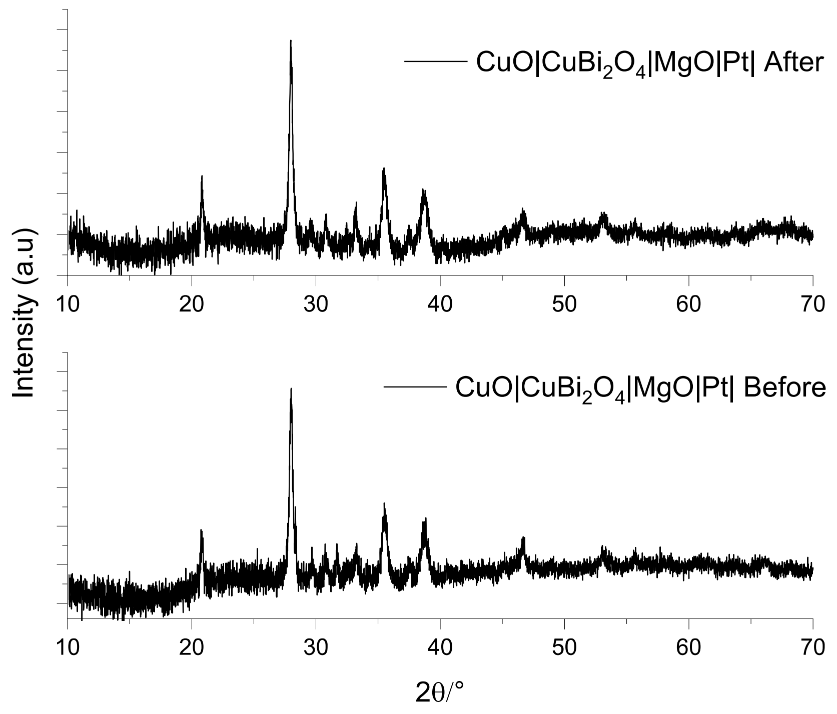

Figure S20 – X-ray diffractogram of CuO|$CuBi_2O_4$|MgO|Pt before and after 3 hours of PEC at 0.4V vs RHE and 1 sun illumination (300W Xe lamp).

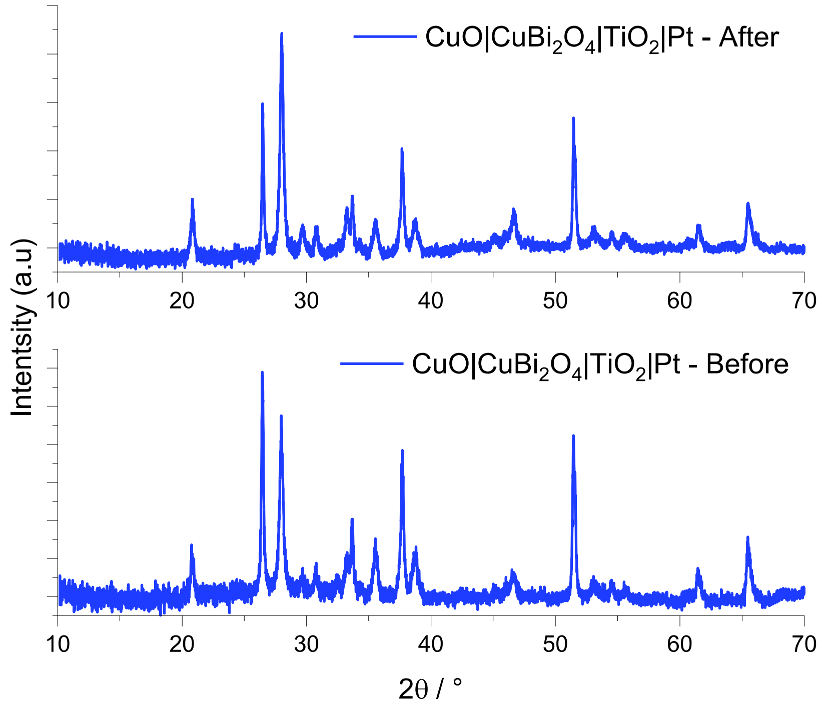

Figure S21 – X-ray diffractogram of CuO|$CuBi_2O_4$|TiO_2|Pt before and after 3 hours of PEC at 0.4V vs RHE and 1 sun illumination (300W Xe lamp).

Additional Tandem Characterization

Transient Absorption Spectroscopy Data

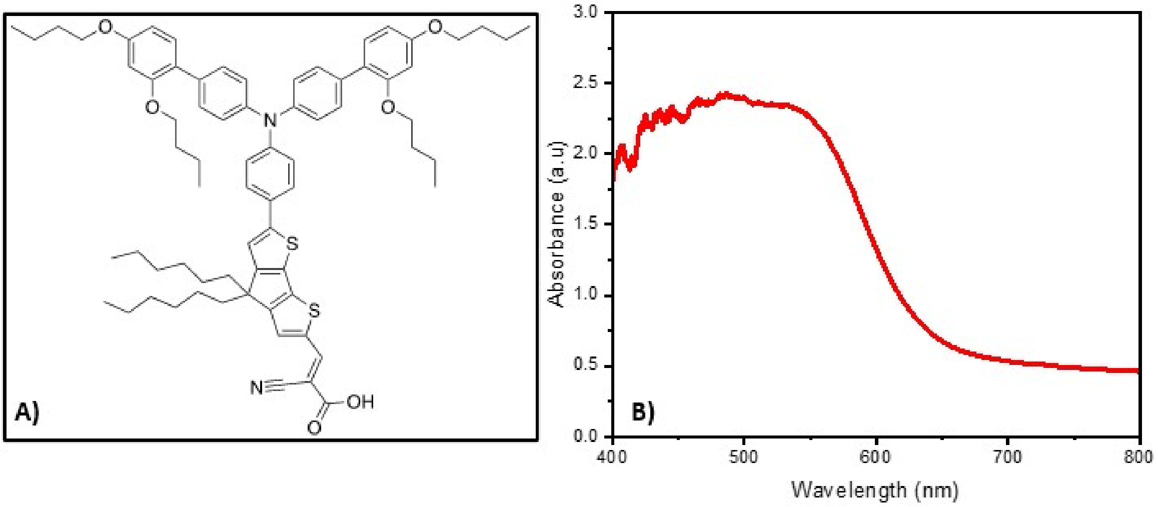

Figure S22 – **A)** Molecular structure of LEG4 dye. **B)** UV-visible absorption spectroscopy of an FTO|TiO_2|LEG4 film.

Sample	Conditions	Y0	A1	t_1 (ps)	A2	t_2 (ps)	A_3	t_3 (ps)
CuO								
	Dry	-0.05±0.39	0.2	9.6±3.2	0.3	114.8±21.8	0.64	3720±499
$CuBi_2O_4$								
	Dry	0.24±0.01	0.26	0.7±0.2	0.296	7.54±0.9	0.192	1490±175
CuO|$CuBi_2O_4$	Electrolyte	0.15±0.01	0.30	19.3±2.1	0.14	124.1±378.0	0.33	1180±185
	Dry	0.15±0.01	0.50	4.8 ±0.4	0.46	944.85	-	-
CuO|$CuBi_2O_4$|TiO_2	Electrolyte	0.15±0.03	0.3	39.9±5.6	0.2	339.6±187.6	0.19	1804±1450
	Dry	0.20±0.01	0.26	5.3±0.6	0.2	71.0±6.3	0.26	1570±145
CuO|$CuBi_2O_4$|NiO	Electrolyte	0.16±0.01	0.27	1.7±0.2	0.4	48.7±3.6	0.2	1180±150
	Dry	0.17±0.03	0.27	18.4±1.4	0.3	334.2±39.3	0.29	3910±1170
CuO|$CuBi_2O_4$|MgO	Electrolyte	0.19±0.01	0.28	2.0±0.2	0.2	53.9±5.4	0.26	1765±157
	Dry	0.14±0.01	0.29	1.5±0.2	0.3	36.3±3.2	0.22	955.2

Table S4 – Table compiling TA lifetimes for CuO, $CuBi_2O_4$, CuO|$CuBi_2O_4$, CuO|$CuBi_2O_4$|TiO_2, CuO|$CuBi_2O_4$|MgO, CuO|$CuBi_2O_4$|NiO. Lifetimes were fitted as exponential decays following the equation: y=a1·exp(x/t1) + a2·exp(x/t2) + a3 ·exp(x/t3)+y0. Y0 is a constant offset or a baseline value, y = change in absorbance, t_x = lifetime component, and A_x = pre-exponential coefficient. Weighted averages of each lifetime is shown in the right-hand column.

Literature Comparison

Photocathode	Applied Potential (vs RHE)	Conditions	Photocurrent Density (mA cm^{-2})	Stable Operation Time (minutes)	Reference
CuO|$CuBi_2O_4$|MgO|Pt	0.4 V	Electrolyte: 0.2 M KCl, 0.01 M H_2KPO_4, and 0.01 M HK_2PO_4 (pH	0.2	180	This work

		7.2). Illumination: 300W Xe lamp calibrated using to 1 sun (filtered with an AM1.5G filter)			
$CuO\|CuBi_2O_4\|TiO_2\|Pt/MoS_2$	0.4 V	As above.	0.1	180	This work
$CuO\|CuBi_2O_4\|NiO\|Pt$	0.3 V	As above.	0.3	180	This work
$CuO\|CuBi_2O_4\|Pt$	0.4 V	As above.	1.2	<10	This work
$CuBi_2O_4$	0.2 V	0.1 M Na_2SO_4	0.45	~3	[1]
Bare $CuBi_2O_4$	0.6 V	0.3 M K_2SO_4 and 0.2 M phosphate buffer (pH 7)	~0.1	300	[2]
Inverse opal $CuBi_2O_4$	0.6 V	K_2SO_4 (0.3 M) and 0.2 M phosphate buffer (pH 6.65) with H_2O_2 as an electron scavenger.	2.95	120	[3]
$CuBi_2O_4$/Au/N, Cu–C	0.5 V	0.3 M K_2SO_4 and 0.2 M Phosphate buffer (pH 6.68)	0.3	50	[4]

$CuBi_2O_4$	0.6 V	0.3 M K_2SO_4 and 0.2 M phosphate buffer (pH 6.65) (With H_2O_2 as an electron scavenger)	~0.97	120	[5]
$CuO\|CuBi_2O_4\|NiO_x$	0.6 V	0.1M NaOH (pH 12.8)	~0.5	300	[6]
$CuO\|CuBi_2O_4$	0 V	0.1 M Na_2SO_4	~0.4	120	[7]
$CuO\|CuBi_2O_4\|Pt$	0.2 V	pH 6.8, 0.3 M K_2SO_4, 0.1 M phosphate, deaerated	0.05	10	[8]

Table S5 – Table detailing a comparison between key electrochemical parameters and stability of homologous systems from the present literature.

References

1. R. Gottesman, A. Song, I. Levine, M. Krause, A. N. Islam, D. Abou-Ras, T. Dittrich, R. van de Krol and A. Chemseddine, *Adv. Funct. Mater.*, **2020**, *30*, 1910832.
2. S. Wei, N. Xu, F. Li, X. Long, Y. Hu, L. Gao, C. Wang, S. Li, J. Ma and J. Jin, *ChemElectroChem*, **2019**, *6*, 3367–3374.
3. D. A. Reddy, Y. Kim, P. Varma, M. Gopannagari, K. A. J. Reddy, D. H. Hong, I. Song, D. P. Kumar and T. K. Kim, *ACS Appl. Energy Mater.*, **2022**, *5*, 6050–6058.
4. N. Nasori, A. Rubiyanto and E. Endarko, *J. Phys.: Conf. Ser.*, **2019**, *1373*, 012016.
5. S. P. Berglund, F. F. Abdi, P. Bogdanoff, A. Chemseddine, D. Friedrich and R. van de Krol, *Chem. Mater.*, **2016**, *28*, 4231–4242.
6. Q. Zhang, B. Zhai, Z. Lin, X. Zhao and P. Diao, *Int. J. Hydrogen Energy*, **2021**, *46*, 11607–11620.
7. S. Pulipaka, N. Boni, G. Ummethala and P. Meduri, *J. Catal.*, **2020**, *387*, 17–27.
8. H. S. Park, C.-Y. Lee and E. Reisner, *Phys. Chem. Chem. Phys.*, **2014**, *16*, 22462–22465.
